# Development and validation of a point‐of‐care nursing mobile tool to guide the diagnosis of malnutrition in hospitalized adult patients: a multicenter, prospective cohort study

**DOI:** 10.1002/mco2.526

**Published:** 2024-04-10

**Authors:** Nan Lin, Xueyan Zhou, Weichang Chen, Chengyuan He, Xiaoxuan Wang, Yuhao Wei, Zhiwen Long, Tao Shen, Lingyu Zhong, Chan Yang, Tingting Dai, Hao Zhang, Hubing Shi, Xuelei Ma

**Affiliations:** ^1^ Department of Biotherapy Cancer Center West China Hospital, Sichuan University Chengdu China; ^2^ Department of Biotherapy State Key Laboratory of Biotherapy, Frontiers Science Center for Disease‐related Molecular Network, West China Hospital, and Key Laboratory of Bio‐Resource and Eco‐Environment of Ministry of Education, College of Life Sciences, Sichuan University Chengdu Sichuan China; ^3^ State Key Laboratory of Oral Diseases National Clinical Research Center for Oral Diseases, Sichuan University Chengdu China; ^4^ Recovery Plus Clinic Chengdu China; ^5^ Department of Colorectal Surgery The Third Affiliated Hospital of Kunming Medical University/Yunnan Tumor Hospital Kunming China; ^6^ Department of Clinical Nutrition Hospital of Chengdu Office of People’s Government of Tibetan Autonomous Region Chengdu China; ^7^ Division of Endocrinology and Metabolism State Key Laboratory of Biotherapy, West China Hospital, Sichuan University Chengdu China; ^8^ Department of Clinical Nutrition West China Hospital, Sichuan University Chengdu China; ^9^ Division of Pancreatic Surgery Department of General Surgery West China Hospital, Sichuan University Chengdu China; ^10^ Laboratory of Integrative Medicine Clinical Research Center for Breast, State Key Laboratory of Biotherapy, West China Hospital, Sichuan University and Collaborative Innovation Center Chengdu Sichuan China

**Keywords:** artificial intelligence, e‐health, facial recognition, malnutrition, mobile multimedia technologies, nutritional screening

## Abstract

Malnutrition is a prevalent and severe issue in hospitalized patients with chronic diseases. However, malnutrition screening is often overlooked or inaccurate due to lack of awareness and experience among health care providers. This study aimed to develop and validate a novel digital smartphone‐based self‐administered tool that uses facial features, especially the ocular area, as indicators of malnutrition in inpatient patients with chronic diseases. Facial photographs and malnutrition screening scales were collected from 619 patients in four different hospitals. A machine learning model based on back propagation neural network was trained, validated, and tested using these data. The model showed a significant correlation (*p* < 0.05) and a high accuracy (area under the curve 0.834–0.927) in different patient groups. The point‐of‐care mobile tool can be used to screen malnutrition with good accuracy and accessibility, showing its potential for screening malnutrition in patients with chronic diseases.

## Graphical abstract

Overview of the proposed model. (A) The procedure of the point‐of‐care mobile solution for screening malnutrition. The device was developed based on various inpatients. Once collecting the basic clinical feature, malnutrition screening scale and facial photograph, the patients’ nutrition status will be shown on mobile phone or computer. (B) The procedure of recruiting participants. (C) The procedure of the development and validation of the face‐eye weighted BP network‐based model.

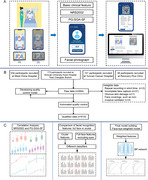

## 1 INTRODUCTION

Malnutrition is a major global issue, with WHO statistics indicating that 828 million people are affected. It has a huge socio‐economic impact on countries, and the diagnosis and treatment of malnutrition is a major challenge. Research has shown that among hospitalized elderly patients in China, the proportion of malnutrition is between 32.3 and 49.8%.^1^ Malnutrition has been linked to a number of negative effects on patients with chronic diseases such as cancer and diabetes.^2^, ^3^ In fact, it is estimated that about 10% to 20% of cancer patients die from malnutrition, not cancer itself.^4^ Additionally, malnourished diabetes patients have a higher occurrence of infection or foot injuries than their well‐nourished peers.^5^ This is a stark reminder of the importance of proper nutrition in order to maintain good health and prevent the onset of serious illnesses.

Early diagnosis can prevent malnutrition and its consequences.^6^ To assess the nutritional status of patients, nutritional scales are typically used. The Patient Generated Subjective Global Assessment (PG‐SGA) is a widely used nutritional assessment and screening tool for cancer patients, incorporating both subjective and objective parameters.^7^ PG‐SGA short form (PG‐SGA SF) was a shortened form of PG‐SGA. Compared as PG‐SGA, PG‐SGA SF are thought easier to be understand and can be completed in short time, but their diagnostic efficiency is close.^8^ The Pearson correlation is calculated as the product of the covariances divided by their standard deviations.^9^ The Nutrition Risk Screening 2002 (NRS2002) is another nutritional risk screening method recommended by the European Society of Parenteral Nutrition (ESPEN) for hospitalized patients.^10^, ^11^ Some objective examination methods were used, such as malnutrition imaging, function, and biomarkers, but these methods are often not in the field due to the economic or practical limitations.^12^ Unfortunately, malnutrition screening is not widely practiced due to lack of awareness or experience in dealing with it. A survey of 20,000 people in 25 European countries found that only 52% of hospitals had a routine program of malnutrition screening on admission.^13^ Thus, hospitals around the world need a set of portable, convenient, accurate and automatic diagnostic tools for screening malnutrition.

The utilization of facial data as a proxy for assessing an individual's body mass index (BMI) has garnered significant attention. In 2010, researchers used 2‐dimensional facial imagery to evaluate BMI, utilizing metrics such as width‐to‐height ratio, perimeter‐to‐area ratio, and cheek‐to‐jaw‐width ratio.^14^, ^15^ Furthermore, more sophisticated algorithms have been developed to automatically extract intricate facial features for predicting BMI or other nutritional indices, such as visceral obesity.^16^, ^17^, ^18^ However, it should be noted BMI is a poor indicator of health outside very specific demographics, thus more specific indicator should be established for screening the malnutrition.

**FIGURE 1:**
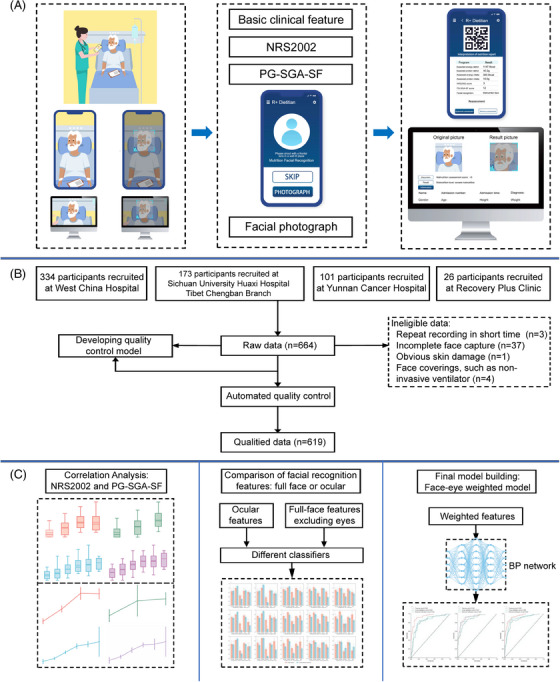
Overview of the proposed model. (A) The procedure of the point‐of‐care mobile tool for screening malnutrition. The device was developed based on various inpatients. Once collecting the basic clinical feature, malnutrition screening scale and facial photograph, the patients’ nutrition status will be shown on mobile phone or computer. (B) The procedure of recruiting participants. (C) The procedure of the development and validation of the face‐eye weighted BP network‐based model.

The development of a digital smartphone‐based self‐administered tool for nutritional risk screening for hospitalized patients has been explored in few studies.^19^ To address this issue, we developed R+ Dietitian, a digital smartphone‐based self‐administered tool. R+ Dietitian enabled us to collect data from four different hospitals in China, including nutritional scales and facial screens.^20^ The aim of this study was to develop and validate a point‐of‐care mobile tool to automatically extract and analyze facial features to screen malnourished patients with chronic disease. We also examined the suitability of NRS2002 and PG‐SGA SF questionnaire, and if any local features, such as eye contour, are more suitable for prediction (Figure 1).

## 2 RESULTS

### 2.1 Characteristics of included participants

We used the R+ Dietitian software to prospectively collect facial data and questionnaire responses from 619 patients. The data collection period was from April 1, 2021 to February 1, 2023. The patients were from West China Hospital in Chengdu (*n* = 329; approval number: 20221153); Sichuan University Huaxi Hospital Tibet Chengban Branch in Chengdu (*n* = 168; approval number: 202273); Yunnan Cancer Hospital in Yunnan (*n* = 96; approval number: SLKYCS2021240); and Recovery Plus Clinic in Chengdu (*n* = 26). The patients were predominantly elderly, with a mean age of 54.64 (SD 14.57), and 61.55% were male. On average, their height was 160.90 (SD 22.18) and weight was 59.22 (SD 11.74), indicating a general nutritional status. The mean NRS2002 score was 1.91 (SD 1.27) and mean PG‐SGA SF score was 5.54 (SD 4.57). Based on the time when recruiting participants, we divided the patients into primary training and validation datasets (*n* = 434), external testing dataset 1 (*n* = 108), and external testing dataset 2 (*n* = 77) (Table 1). No statistically significant findings between primary training and validation datasets and external testing dataset 1. Because of the external testing dataset 2 only included cancer patients, some of the indices like PG‐SGA SF score were found to contain statistical difference between groups.

**TABLE 1:** Basic information about included participants.

Characteristics	Primary training and validation datasets (*n* = 434)	External testing dataset 1 (*n* = 108)	External testing dataset 2 (*n* = 77)	*p* Value
Dataset				
West China Hospital	197(45.39%)	55(50.93%)	77(100.00%)	
Sichuan University Huaxi Hospital Tibet Chengban Branch	142(32.72%)	26(24.07%)	0(0.00%)	
Yunnan Cancer Hospital	76(17.51%)	20(18.52%)	0(0.00%)	
Recovery Plus Clinic	19(4.38%)	7(6.48%)	0(0.00%)	
Types of disease				
Cancer inpatient^a^	230(53.00%)	63(58.33%)	77(100.00%)	
Other inpatient^b^	184(42.40%)	40(37.04%)	0(0.00%)	
Normal control^c^	20(4.61%)	5(4.63%)	0(0.00%)	
Gender				0.396
Male	267(61.52%)	71(65.74%)	43(55.84%)	
Female	167(38.48%)	37(34.26%)	34(44.16%)	
Age (mean ± SD)	53.66 ± 15.71	51.98 ± 15.56	58.35 ± 12.29	0.016
Weight (mean ± SD)	60.74 ± 14.96	60.36 ± 12.93	57.63 ± 9.69	0.203
Height (mean ± SD)	164.45 ± 10.78	165.15 ± 8.26	160.99 ± 8.21	0.011
NRS2002 score (mean ± SD)	1.88 ± 1.27	2.12 ± 1.30	1.79 ± 1.24	0.140
PG‐SGA SF score (mean ± SD)	4.53 ± 3.04	5.19 ± 3.54	6.57 ± 5.58	0.001

^a^ Including patients diagnosed as malignant tumor, such as lung cancer, breast cancer, esophageal cancer, and so on.

^b^ Including patients who are not diagnosed as malignant tumors but still need treatment as inpatient, such as type 2 diabetes, hypertension, heart failure, bone fracture, and so on.

^c^ Including participants who are not inpatients but medical staffs without basic diseases.

### 2.2 Classification criteria and feasibility analysis

We conducted a Pearson correlation analysis to determine the correlation between the scores obtained from the two systems. The analysis showed a significant correlation (*p *< 0.05; Table S1) in all groups. In the real world, the NRS2002 threshold score of three indicates potential malnutrition risk, while the PG‐SGA SF threshold scores of 2, 4, and 9 indicate normal nutrition status, potential risk, need for nutritional intervention, and severe malnutrition, respectively. Our subsequent analysis of the distribution of scores (Figures S1 and S2) revealed that the mean value of PG‐SGA SF corresponding to the NRS2002 threshold score of 3.0 was 5.079, similar to the PG‐SGA SF threshold score of 4.0, indicating the need for nutritional intervention. Furthermore, the mean value of PG‐SGA SF corresponding to the NRS2002 threshold score of 0.0 was 2.562, indicating good nutrition, which is similar to the PG‐SGA SF threshold score of 2.0. Based on these findings, we eventually chose the PG‐SGA SF scores of 2.0 and 5.0 as the three classification thresholds, representing normal nutritional status, malnutrition, and severe malnutrition.^21^

### 2.3 Comparison of multiple models with different features

After five‐class classification models and 50 rounds of training (Table S2), the results showed that the model using ocular features achieved higher area under the curve (AUC) values than the model using full‐face features excluding eyes in the external verification set (with the exception of KNN in the external verification cohort of Other Inpatient Group; Table 2 and Figure S3), higher accuracy (with the exceptions of XGBoost in the training cohort of Cancer Inpatient Group under the training cohort and Extratree in the external verification cohort of Other Inpatient Group; Table 2 and Figure S4), as well as higher sensitivity (with exceptions of Adaboost in the Other Inpatient Group; Table 2 and Figure S4).

**TABLE 2:** Comparison of the use of ocular features and full‐face features excluding eyes in the PG‐SGA SF system.

		Ocular features					Full‐face features excluding eyes			
Group	Algorithm	Cohort	Accuracy	Sensitivity	Specificity	AUC	Accuracy	Sensitivity	Specificity	AUC
Cancer Inpatient Group	Adaboost	Training cohort	0.708 (0.611–0.806)	0.826 (0.780–0.872)	0.500 (0.317–0.683)	0.726 (0.664–0.789)	0.611 (0.442–0.781)	0.857 (0.805–0.909)	0.267 (0.147–0.386)	0.672 (0.622–0.721)
		Cross validation cohort	0.667 (0.498–0.835)	0.750 (0.691–0.809)	0.536 (0.482–0.590)	0.778 (0.735–0.820)	0.611 (0.453–0.770)	0.732 (0.669–0.794)	0.452 (0.279–0.625)	0.678 (0.632–0.723)
		External verification cohort	0.694 (0.590–0.799)	0.812 (0.754–0.871)	0.458 (0.383–0.533)	0.719 (0.646–0.791)	0.681 (0.628–0.733)	0.860 (0.819–0.902)	0.414 (0.332–0.496)	0.652 (0.593–0.711)
	Extratree	Training cohort	0.583 (0.474–0.693)	0.609 (0.522–0.695)	0.538 (0.406–0.671)	0.725 (0.660–0.789)	0.583 (0.522–0.644)	0.681 (0.627–0.734)	0.400 (0.282–0.518)	0.676 (0.627–0.724)
		Cross validation cohort	0.625 (0.552–0.698)	0.769 (0.695–0.844)	0.455 (0.311–0.598)	0.736 (0.692–0.779)	0.625 (0.521–0.729)	0.619 (0.580–0.658)	0.633 (0.464–0.803)	0.655 (0.582–0.729)
		External verification cohort	0.625 (0.569–0.681)	0.651 (0.581–0.722)	0.586 (0.446–0.726)	0.735 (0.683–0.787)	0.597 (0.511–0.684)	0.902 (0.853–0.952)	0.194 (0.066–0.321)	0.632 (0.569–0.696)
	RF	Training cohort	0.625 (0.500–0.750)	0.868 (0.768–0.969)	0.353 (0.271–0.435)	0.724 (0.658–0.789)	0.528 (0.432–0.623)	0.582 (0.509–0.655)	0.353 (0.267–0.439)	0.614 (0.539–0.688)
		Cross validation cohort	0.625 (0.552–0.698)	0.725 (0.635–0.815)	0.500 (0.332–0.668)	0.709 (0.634–0.784)	0.542 (0.480–0.603)	0.630 (0.522–0.739)	0.385 (0.253–0.516)	0.615 (0.558–0.673)
		External verification cohort	0.583 (0.454–0.713)	0.667 (0.563–0.770)	0.444 (0.331–0.557)	0.663 (0.594–0.733)	0.583 (0.476–0.691)	0.610 (0.574–0.645)	0.548 (0.385–0.711)	0.644 (0.577–0.712)
	Xgboost	Training cohort	0.583 (0.519–0.647)	0.673 (0.623–0.724)	0.391 (0.334–0.448)	0.776 (0.732–0.821)	0.611 (0.454–0.768)	0.667 (0.563–0.771)	0.533 (0.474–0.593)	0.696 (0.630–0.762)
		Cross validation cohort	0.639 (0.558–0.720)	0.690 (0.640–0.741)	0.567 (0.394–0.740)	0.690 (0.629–0.752)	0.597 (0.441–0.754)	0.682 (0.579–0.784)	0.464 (0.306–0.623)	0.685 (0.629–0.740)
		External verification cohort	0.597 (0.509–0.686)	0.647 (0.543–0.751)	0.476 (0.326–0.627)	0.715 (0.674–0.755)	0.556 (0.387–0.724)	0.641 (0.592–0.690)	0.455 (0.287–0.622)	0.603 (0.537–0.669)
	KNN	Training cohort	0.681 (0.586–0.775)	0.820 (0.759–0.881)	0.364 (0.251–0.476)	0.723 (0.663–0.783)	0.611 (0.516–0.706)	0.698 (0.599–0.798)	0.368 (0.253–0.483)	0.674 (0.603–0.745)
		Cross validation cohort	0.625 (0.464–0.786)	0.744 (0.705–0.782)	0.485 (0.400–0.570)	0.679 (0.634–0.723)	0.597 (0.451–0.743)	0.714 (0.623–0.805)	0.348 (0.288–0.408)	0.659 (0.609–0.708)
		External verification cohort	0.694 (0.601–0.788)	0.891 (0.824–0.959)	0.346 (0.182–0.510)	0.707 (0.666–0.748)	0.639 (0.530–0.748)	0.769 (0.669–0.870)	0.485 (0.347–0.622)	0.683 (0.616–0.750)
Other Inpatient Group	Adaboost	Training cohort	0.767 (0.654–0.879)	0.872 (0.782–0.962)	0.571 (0.411–0.732)	0.842 (0.781–0.904)	0.683 (0.604–0.763)	0.694 (0.661–0.728)	0.667 (0.578–0.756)	0.663 (0.617–0.708)
		Cross validation cohort	0.733 (0.583–0.884)	0.814 (0.778–0.850)	0.529 (0.416–0.642)	0.845 (0.799–0.891)	0.617 (0.489–0.745)	0.684 (0.584–0.784)	0.500 (0.428–0.572)	0.607 (0.559–0.655)
		External verification cohort	0.767 (0.697–0.836)	0.824 (0.768–0.879)	0.692 (0.569–0.815)	0.794 (0.733–0.854)	0.683 (0.615–0.752)	0.810 (0.753–0.866)	0.389 (0.312–0.466)	0.669 (0.624–0.715)
	Extratree	Training cohort	0.767 (0.639–0.894)	0.800 (0.703–0.897)	0.700 (0.643–0.757)	0.837 (0.798–0.876)	0.700 (0.593–0.807)	0.868 (0.804–0.933)	0.409 (0.317–0.501)	0.693 (0.637–0.750)
		Cross validation cohort	0.817 (0.741–0.892)	0.825 (0.747–0.903)	0.800 (0.750–0.850)	0.846 (0.777–0.914)	0.717 (0.556–0.877)	0.756 (0.709–0.803)	0.632 (0.546–0.717)	0.802 (0.756–0.849)
		External verification cohort	0.667 (0.519–0.814)	0.800 (0.709–0.891)	0.533 (0.360–0.707)	0.702 (0.651–0.752)	0.683 (0.551–0.815)	0.757 (0.665–0.849)	0.565 (0.477–0.653)	0.771 (0.703–0.838)
	RF	Training cohort	0.700 (0.550–0.850)	0.805 (0.751–0.859)	0.474 (0.402–0.546)	0.791 (0.746–0.836)	0.633 (0.541–0.725)	0.758 (0.650–0.865)	0.481 (0.321–0.642)	0.701 (0.643–0.759)
		Cross validation cohort	0.683 (0.628–0.739)	0.762 (0.719–0.805)	0.500 (0.409–0.591)	0.777 (0.738–0.816)	0.700 (0.580–0.820)	0.810 (0.704–0.916)	0.444 (0.310–0.579)	0.737 (0.702–0.773)
		External verification cohort	0.700 (0.575–0.825)	0.732 (0.680–0.784)	0.632 (0.524–0.739)	0.766 (0.709–0.824)	0.567 (0.430–0.704)	0.645 (0.539–0.751)	0.483 (0.345–0.620)	0.623 (0.572–0.673)
	Xgboost	Training cohort	0.700 (0.536–0.864)	0.839 (0.739–0.938)	0.552 (0.438–0.665)	0.770 (0.722–0.818)	0.667 (0.615–0.718)	0.750 (0.671–0.829)	0.500 (0.328–0.672)	0.751 (0.698–0.805)
		Cross validation cohort	0.700 (0.644–0.756)	0.825 (0.780–0.870)	0.450 (0.390–0.510)	0.784 (0.731–0.837)	0.633 (0.546–0.720)	0.641 (0.569–0.713)	0.619 (0.465–0.773)	0.707 (0.643–0.771)
		External verification cohort	0.600 (0.500–0.700)	0.659 (0.550–0.767)	0.474 (0.304–0.643)	0.716 (0.668–0.763)	0.583 (0.482–0.685)	0.659 (0.615–0.702)	0.421 (0.354–0.488)	0.662 (0.597–0.726)
	KNN	Training cohort	0.783 (0.622–0.944)	0.868 (0.780–0.957)	0.636 (0.534–0.738)	0.819 (0.779–0.859)	0.667 (0.508–0.825)	0.714 (0.620–0.808)	0.556 (0.476–0.635)	0.705 (0.646–0.763)
		Cross validation cohort	0.750 (0.589–0.911)	0.850 (0.760–0.940)	0.550 (0.470–0.630)	0.807 (0.753–0.862)	0.583 (0.531–0.636)	0.800 (0.721–0.879)	0.280 (0.097–0.463)	0.644 (0.583–0.705)
		External verification cohort	0.683 (0.526–0.840)	0.879 (0.828–0.930)	0.444 (0.301–0.588)	0.746 (0.678–0.815)	0.733 (0.617–0.849)	0.868 (0.816–0.921)	0.500 (0.412–0.588)	0.756 (0.720–0.793)
All Inpatient Group	Adaboost	Training cohort	0.655 (0.523–0.787)	0.797 (0.695–0.899)	0.460 (0.366–0.554)	0.753 (0.705–0.802)	0.672 (0.509–0.836)	0.882 (0.817–0.947)	0.302 (0.138–0.467)	0.698 (0.643–0.753)
		Cross validation cohort	0.672 (0.609–0.735)	0.795 (0.757–0.833)	0.389 (0.319–0.459)	0.740 (0.690–0.791)	0.639 (0.541–0.736)	0.912 (0.881–0.944)	0.077 (0.014–0.140)	0.680 (0.635–0.725)
		External verification cohort	0.655 (0.499–0.812)	0.829 (0.752–0.906)	0.349 (0.172–0.525)	0.747 (0.695–0.798)	0.630 (0.461–0.800)	0.890 (0.828–0.952)	0.217 (0.121–0.314)	0.662 (0.622–0.702)
	Extratree	Training cohort	0.647 (0.525–0.769)	0.811 (0.711–0.911)	0.378 (0.231–0.524)	0.754 (0.694–0.814)	0.664 (0.527–0.801)	0.936 (0.905–0.967)	0.146 (0.029–0.264)	0.726 (0.679–0.774)
		Cross validation cohort	0.731 (0.658–0.804)	0.840 (0.766–0.914)	0.545 (0.490–0.601)	0.792 (0.750–0.834)	0.639 (0.508–0.770)	0.946 (0.911–0.980)	0.133 (0.011–0.256)	0.701 (0.640–0.761)
		External verification cohort	0.706 (0.538–0.874)	0.913 (0.882–0.944)	0.420 (0.355–0.485)	0.771 (0.726–0.815)	0.605 (0.464–0.747)	0.919 (0.883–0.955)	0.099 (0.023–0.175)	0.636 (0.587–0.685)
	RF	Training cohort	0.739 (0.641–0.838)	0.813 (0.780–0.846)	0.614 (0.439–0.788)	0.790 (0.723–0.858)	0.681 (0.518–0.844)	0.868 (0.821–0.916)	0.349 (0.252–0.446)	0.695 (0.657–0.733)
		Cross validation cohort	0.723 (0.584–0.861)	0.886 (0.855–0.916)	0.490 (0.307–0.673)	0.763 (0.725–0.801)	0.605 (0.467–0.743)	0.775 (0.702–0.847)	0.354 (0.214–0.494)	0.617 (0.555–0.678)
		External verification cohort	0.689 (0.573–0.805)	0.877 (0.809–0.945)	0.463 (0.363–0.562)	0.731 (0.664–0.798)	0.538 (0.421–0.655)	0.776 (0.674–0.878)	0.231 (0.075–0.386)	0.598 (0.547–0.649)
	Xgboost	Training cohort	0.739 (0.647–0.832)	0.833 (0.730–0.937)	0.561 (0.435–0.687)	0.752 (0.688–0.816)	0.664 (0.598–0.729)	0.759 (0.712–0.805)	0.406 (0.223–0.589)	0.713 (0.640–0.786)
		Cross validation cohort	0.782 (0.665–0.898)	0.873 (0.792–0.955)	0.600 (0.462–0.738)	0.785 (0.711–0.858)	0.672 (0.605–0.740)	0.805 (0.715–0.895)	0.378 (0.237–0.520)	0.693 (0.656–0.729)
		External verification cohort	0.689 (0.606–0.772)	0.806 (0.728–0.883)	0.511 (0.343–0.678)	0.741 (0.670–0.812)	0.647 (0.483–0.812)	0.740 (0.675–0.805)	0.476 (0.324–0.628)	0.647 (0.579–0.715)
	KNN	Training cohort	0.706 (0.612–0.799)	0.789 (0.684–0.895)	0.558 (0.384–0.733)	0.825 (0.758–0.892)	0.630 (0.498–0.763)	0.778 (0.696–0.859)	0.404 (0.270–0.538)	0.711 (0.674–0.749)
		Cross validation cohort	0.697 (0.551–0.844)	0.928 (0.886–0.969)	0.380 (0.208–0.552)	0.788 (0.727–0.850)	0.597 (0.448–0.745)	0.754 (0.701–0.807)	0.380 (0.231–0.529)	0.668 (0.608–0.728)
		External verification cohort	0.639 (0.559–0.719)	0.765 (0.725–0.805)	0.324 (0.218–0.429)	0.733 (0.666–0.799)	0.647 (0.498–0.796)	0.747 (0.656–0.837)	0.477 (0.382–0.573)	0.709 (0.660–0.757)

Data are mean (95% CI) fivefold cross‐validation method was applied in each testing dataset. AUC, area under the receiver operating characteristic curve.

### 2.4 Development of face‐eye weighted model

**FIGURE 2:**
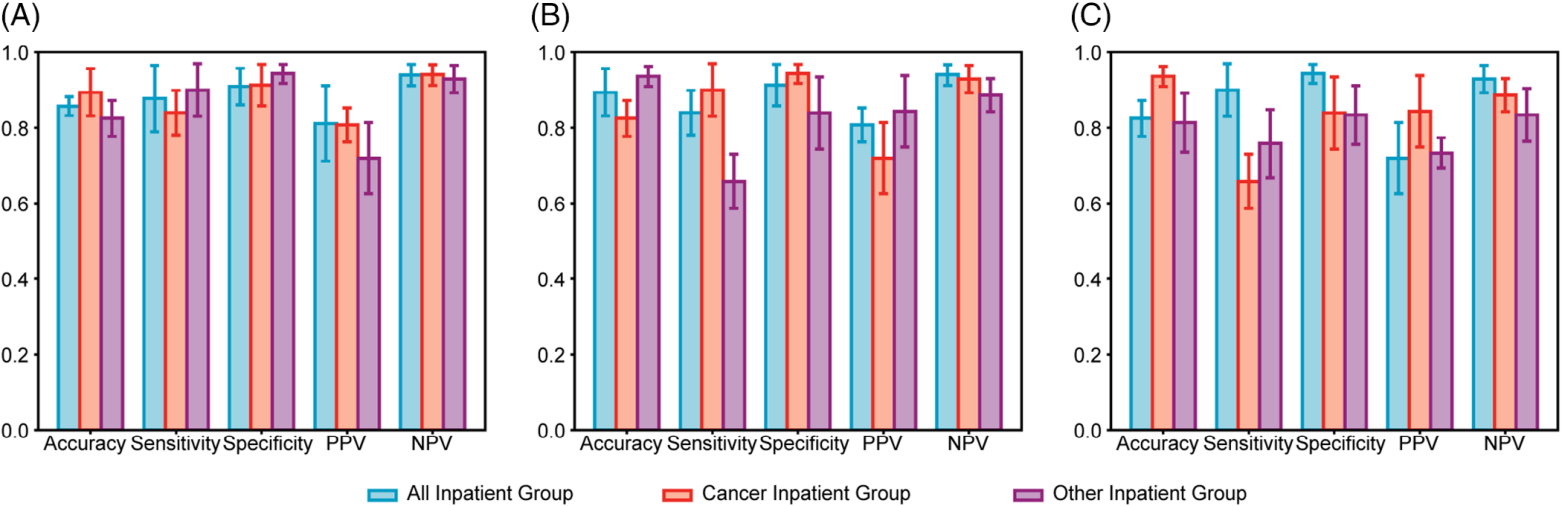
Performance of feature‐weighted model to classify nutritional status of different inpatient groups. Feature‐weighted model was used in Cancer Inpatient Group (A), Other Inpatient Group (B), and All Inpatient Group (C) to classify nutrition status. The bar chart was used to compare the five parameters of Accuracy, Sensitivity, Specificity, PPV, and NPV. Bar represents the average value, and the distance between the upper boundary and the lower boundary of the error bar and bar is the corresponding 95% CI. CI, confidence interval; NPV, negative predictive value; PPV, positive predictive value; RF, random forest.

**FIGURE 3:**
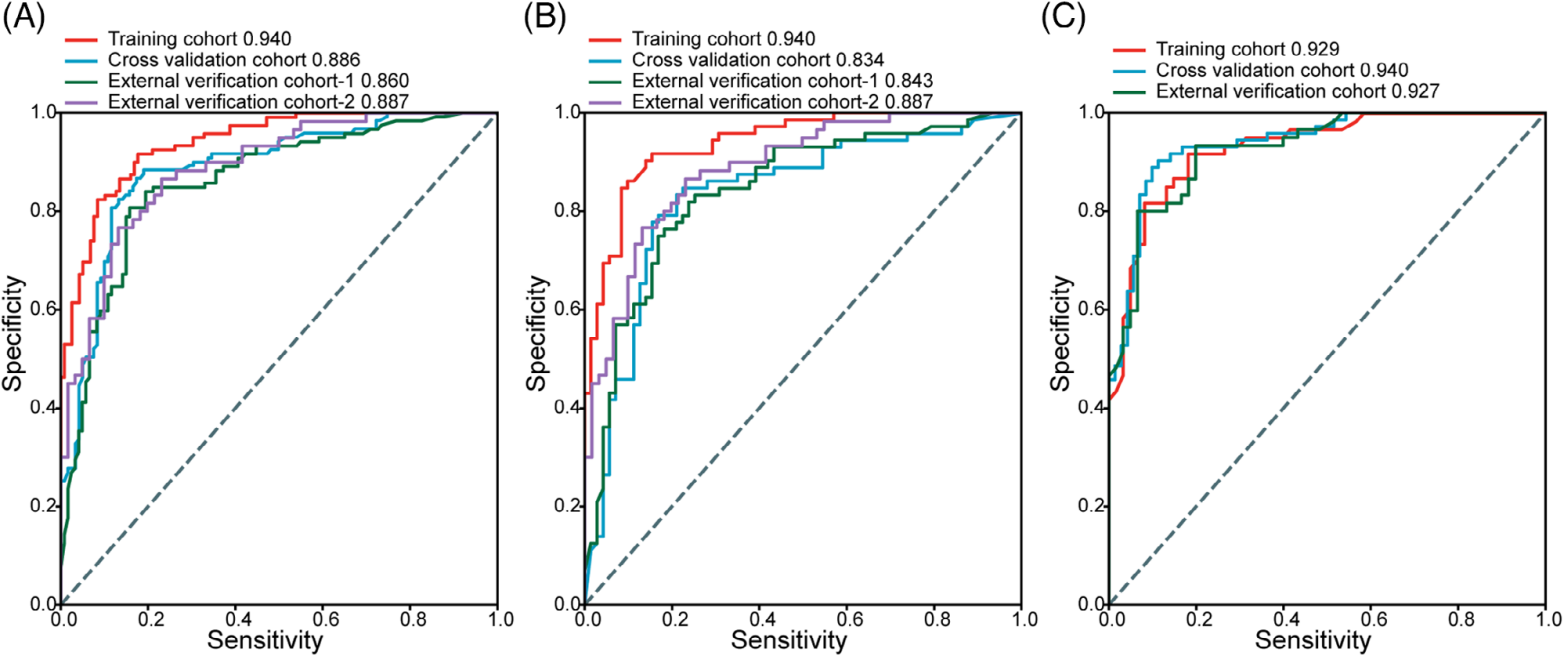
Performance of feature‐weighted model to classify nutritional status of different inpatient groups. Feature‐weighted model was used in All Inpatient Group (A), Cancer Inpatient Group (B), and Other Inpatient Group to classify nutrition status. Compare the AUC results of the training cohort, cross validation cohort and external validation cohort using different features under the different inpatient groups through the ROC curve. The AUC is micro‐AUC. AUC, area under the curve; RF, random forest; ROC, receiver operating characteristic.

Based on the face‐eye weighted features, we constructed a back propagation (BP) neural network model, which we evaluated on the Cancer Inpatient Group, Other Inpatient Group, and All Inpatient Group using fivefold cross‐validation and an external verification set. The average AUC (Figure 2) obtained from the fivefold cross‐validation set was 0.886 (CI 0.843–0.930), 0.834 (CI 0.764–0.904), and 0.927 (CI 0.899–0.955) for the Cancer Inpatient Group, Other Inpatient Group, and All Inpatient Group, respectively, with the corresponding AUC obtained from the external verification set being 0.860 (CI 0.817–0.904), 0.843 (CI 0.796–0.889), and 0.887 (CI 0.829–0.944). The negative predictive value (NPV) and positive predictive value (PPV) for all three groups were not less than 0.75, the sensitivity was not less than 0.8, and the Specificity was not less than 0.6, except for the Other Inpatient Group, where the Accuracy was 0.778 (CI 0.642–0.913), but still not less than 0.8 in the other two groups (Figure 3 and Table S3).

### 2.5 Statistical analysis

To further validate our model's superiority, we conducted DeLong test,^22^ Net Reclassification Improvement (NRI) test^23^ and Integrated Discrimination Improvement (IDI) test.^24^ By comparing the results of the same model with different features, we found that the training results of the model using ocular features were better than those using full‐face features excluding eyes (*p *< 0.05). For face‐eye weighted model, we found that it was better than the nonweighted model in identifying malnutrition classification in these tests (*p *< 0.05).

### 2.6 Platforms establishment

We developed a system platform and a WeChat mini program called Malnutrition Screening Eye (MSE), adaptable to both computers and mobile phones, based on the model we developed (Figure S5). This mini‐program provides clinical data collection functionality by allowing users to fill in their height, weight, disease type, weight change over time, appetite change over time, food intake change over time, dietary habits, and intake of various foods. It also enables facial data collection by capturing real‐time facial images through the phone camera. Based on the embedded algorithms, it achieves nutritional recognition based on facial expressions. The program provides real‐time evaluation results on users' energy deficit, expected protein deficit, energy intake, protein intake, NRS2002 score, PG‐SGA SF score, and facial recognition results. Additionally, it offers users dietary task recommendations and a historical evaluation query function. This mini‐program is compatible with both IOS and Android platforms.

## 3 DISCUSSION

This study aimed to develop and validate a malnutrition screening tool called the MSE in several Chinese hospitals to evaluate its suitability for assessing malnutrition in inpatients. Traditionally, nutritional assessment relied on user‐based properties such as NRS 2002 and PG‐SGA SF questionnaires. In contrast, MSE utilizes automated facial expression analysis to automatically detect malnutrition using eye attention enhancement, which shortens the malnutrition assessment to three seconds in real‐world settings. Additionally, MSE can be operated from a mobile device, offering potential benefits for long‐term nutritional information management of patients. The combination of these properties offers a new option for doctors and patients to improve their nutritional assessment workflow, potentially leading to changes in clinical practice to address current challenges around malnutrition screening in areas without specialized dietitians. Furthermore, to validate the superiority of MSE, the study conducted the net reclassification and IDI test, which showed it was better than the nonweighted model in identifying pain classification (*p *< 0.05; Figure S4). Finally, a system platform called MSE was developed, adaptable to both computers and mobile phones, based on the model developed (Figures S5 and S6).

It is worth noting that during physical assessments conducted using the PG‐SGA SF, healthcare professionals typically only evaluate the orbital fat pad and temporal muscle in the facial region.^7^, ^8^ The morphology of the periorbital region changes significantly in the case of tissue loss and is largely determined by the volume of the periorbital fat pads.^25^ These comprise the temporal fat pad, the preaponeurotic fat pad, the nasal fat pads, and the central fat pad. In cases of lipoatrophy, the loss of volume in these fat pads has been observed to accentuate the superior, medial, and lateral edges of the orbital rim, contributing to a sunken, skeletonized appearance.^26^ Due to the limitations of camera‐captured images in providing depth information, we made the decision to use the orbital region and the rest of the face excluding the orbital region as two distinct features for inclusion in our study. Our analysis indicated that using the periorbital region as a feature yielded superior results with the same model. In other studies, such as the identification of fatigue in natural environments, the performance of the eye model has also been shown to be superior to that of the face model, showing its potential unsegment.^27^

To ensure the reliability and robustness of our model, we conducted extensive analysis on patients from multiple centers and types. In the training set and the first external verification, we included multiple types of patients, such as cancer or diabetes, to enhance the applicability and universality of our model. Meanwhile, the second external verification set only included cancer patients, limiting the scope of our model but providing valuable guidance and inspiration for research on different types of patients. For cross‐validation, we evaluated the performance of our model by dividing the data into five groups, using four groups for training and one group for validation. This method effectively reduced the risk of overfitting and improved the model's robustness.^28^ Bootstrap, a nonparametric method that does not require assumptions about the distribution of the data, was used to calculate the confidence intervals (CIs) of multiple evaluation metrics for the external verification set.^29^ This approach allowed us to comprehensively evaluate the performance of the model and improve the reliability of the evaluation results. In comparing models, we employed Delong test, IDI test, and NRI test to handle differences in receiver operating characteristic (ROC) curve shape, sample size, and other factors, providing a more objective and intuitive comparison result. The *p* value obtained by Delong‐test could be used for significance testing to determine if the difference between two models was significant.^30^

With the development of e‐health, more and more point‐of‐care health app were developed and used to help medical stuffs and patients to get more convenient survice.^31^, ^32^ In the field of monitoring hypertension, study have found 25 research, while two of them have proposed a contactless approach based on facial videos.^33^, ^34^ In the fields of procedural pain and dementia, studies also revealed the potential of using unsegmented facial‐based data.^35^, ^36^ In the other field of diagnosing genetic diseases, facial‐photograph‐based e‐health technology has also showed it potential.^37^, ^38^ In the field of screening malnutrition, more and more mobile phone interventions were developed.^39^, ^40^ These studies helped to screen malnutrition especially in low‐ and middle‐income countries, but were also limited by low network capacity and limited access to mobile phones to specific technical barriers.^41^, ^42^, ^43^ Malnutrition screening is a very important link in inpatients with various disease to improve their outcomes.^44^ Since the malnutrition due to various diseases has a certain performance on the patient's face, we developed and validated a malnutrition screening tool for multiple diseases. Our screening tool successfully makes the evaluation of nutritional status objective and visual, while simplifying the assessment and monitoring process for clinical doctors and nursing staff. In addition, the WeChat mini‐program is compatible with various mobile operating systems, making it highly portable and accessible.^20^, ^45^

There are some limitations that need to be addressed. First, the validation of the model was only conducted on tumor patients in the second external verification set, and other types of patients were not included. Furthermore, the accuracy of the model may be affected by the lighting and shooting angle, which requires image quality screening and restrictions. Additionally, the universality and applicability of the model may be limited by factors such as race and age, and thus should be taken into consideration during future development. Moreover, although the amount of data used in the study has been increased compared with previous works, it is still relatively small, and only machine learning was employed. Therefore, further research is required to expand the data scale, collect more video images, and apply more advanced methods such as deep learning to enhance the accuracy and stability of the model.

## 4 CONCLUSION

In summary, we developed, validated, and tested a facial photograph‐based machine point‐of‐care platform for malnutrition screening with high applicability and universality. Our proof‐of concept study provides a unique and generalizable model that could be used in community settings to screen for malnutrition in inpatients with multiple diseases.

## 5 MATERIALS AND METHODS

### 5.1 Overview of the study

In this prospective, multicenter, cohort study, we aimed to develop point‐of‐care mobile tool to assist in the diagnosis of malnutrition during the intake prior to examination by a nurse or doctor. To achieve this goal, we divided the research into several individual parts. First, we evaluate the correlation between PG‐SGA SF and NRS2002 to determine whether these two methods can be used as a predictor of patient nutrition, and which scale should be chosen. Next, we utilized a variety of machine learning models to explore whether the ocular area could serve as an enhanced region for facial recognition nutrition. Therefore, we proposed and validated a vision‐based eye‐face weighted model using BP neural network. Finally, we developed a system platform and a WeChat mini program called MSE, adaptable to both computers and mobile phones.

### 5.2 Ethics and inclusion statement

The inclusion criteria were: (1) were 18 years or older and had the cognitive ability to complete a questionnaire and (2) were able to give informed consent. The exclusion criteria were (1) condition was too severe to complete the questionnaire and (2) have obvious skin lesions on the face that prevented face recognition. All participants provided informed written consent to complete the nutritional evaluation and facial photograph used for research and development purposes. The research was registered in the Chinese Clinical Trial Registry (ChiCTR) with registration number ChiCTR2200063512. Informed consent statement was gained from all of the patients.

### 5.3 Procedures

R+ Dietitian was a mobile‐phone based mini program designed to provide nutritional risk screening and dietary assessment. In a previous study, it was compared with experienced dietitians, and found to have high accuracy, sensitivity, and specificity.^20^ This mini program collects basic information from patients, and screens their nutritional risk. After the assessment, participants can choose whether to take a picture to provide their face data for analysis. In the four hospitals included in this research, we licensed the program to doctors or nurses to use it as a daily nutritional assessment tool. The study participants were divided into cancer inpatients, other inpatients, and normal controls based on their medical condition. Those with noticeable skin damage were excluded from the study. The detailed procedure is described in the Supplementary Material. After completing the participants’ daily nutritional assessment, doctors or nurses instructed the patients to take facial photos and explained the questionnaire results to them. The detailed procedure of this research was shown in the Supplementary Material.

### 5.4 Correlation between PG‐SGA SF and NRS2002

To demonstrate the applicability of the nutrient classification method explored in this study, we analyzed the correlation between PG‐SGA SF and NRS2002. The NRS‐2002 tool is recommended by the ESPEN to screen for nutritional risk in the hospital settings, while the PG‐SGA SF is typically used to screen for nutritional risk in patients with cancer.^46^, ^47^, ^48^

### 5.5 Face detection and correction

For subsequent experiments, we performed face detection and correction on the image. Due to the nonuniform shooting pose and the presence of facial occlusion, we used OpenCV to detect and correct faces in the images to eliminate these errors. We used the Face_recognition algorithm to detect 68 facial key points, including the face contour, eyebrow arch, eye contour, nose, and mouth contour. After obtaining the coordinates of the key points, we calculated the center of the left and right eyes, respectively. We then took the line between the two centers to calculate the angle *θ* with the horizontal direction and rotated the image counterclockwise by *θ*, with the rotation center being the overall center of the face. For ease of analysis, we cropped and size‐normalized each image. The cropping criterion was as follows: we divided the rotated image into top, middle, and bottom parts based on the facial key points, with the middle part's height being 40% of the distance from the center coordinates of the left and right eyes to the overall center coordinates of the mouth. The top and bottom heights were 30%, and the width was equal to the sum of the heights of the three parts. We fixed the image size at 256 × 256.

### 5.6 Feature extraction and dimensionality reduction

We utilized the Histogram of Oriented Gradients (HOG) descriptor to extract features, which constructs global features using local statistics of image gradient edge directions. First, we normalized the image color space and adjusted the image contrast using a Gamma value of 2.2 to eliminate the influence of shadow and illumination difference. We then calculated the gradient information of each pixel in the image, which further reduced the illumination interference while capturing the contour information. Next, we set the cell size to 6 × 6, the bin size to 8, and the angle range corresponding to each bin to 45°. We calculated the gradient direction information of each cell based on the gradient information of each image pixel, and finally concatenated the feature vectors of all cells together to form the features of the entire image. To reduce the influence of the variation range of the gradient intensity, we normalized the gradient intensity of the block before performing series summation. Finally, we used principal component analysis to perform dimensionality reduction on the obtained features.

### 5.7 Comparison of multiple models

We used Adaboost, Extratree, random forest (RF), Xgboost, and KNN models to compare the classification performance of full‐face features excluding eyes and ocular features in the PG‐SGA SF system for facial assessment section. To crop the ocular features region, we performed face detection and correction, and obtained 68 facial landmarks. Then, we selected the region of the eye feature using the leftmost point of the left eyebrow as the upper‐left coordinate, the rightmost point of the right eyebrow as the upper‐right coordinate, and the third point of the nose as the height. For cropping the full‐face features excluding eyes, we directly cropped the region of the eye feature from the image after face detection and correction.

### 5.8 Face‐eye weighted model development

To enhance the classification performance of our model, we utilized a facial area segmentation and weighted approach to retain the information of full‐face features. Specifically, we first divided the cropped facial image into 6 × 6 blocks and extracted their HOG features. Then, based on the position information of facial landmarks, we determined whether each block belonged to the eye region and assigned four times the weight if it did, otherwise multiplied by one time the weight. Finally, we trained the model using a BP neural network with stochastic gradient descent optimization.

### 5.9 Statistical analysis

To investigate the distribution of the included patient population, we utilized the average value of PG‐SGA SF corresponding to NRS2002 as a result indicator and computed various statistical measures such as the median, upper quartile, lower quartile, maximum, minimum, and 95% CI. To assess the correlation between the included population PG‐SGA SF and NRS2002, we employed Pearson test and used a correlation coefficient as an evaluation indicator. We considered *p*‐values less than 0.05 to indicate statistical significance. To evaluate the classification performance of various models, we utilized six metrics, including accuracy, sensitivity, specificity, PPV, NPV, and AUC, and computed the 95% CI for each indicator. Bootstrap method was used to determine the 95% CI of the external verification cohort. In order to facilitate the comparison of ROC curves obtained by different models under the same training cohort, we conducted DeLong test, IDI test, and NRI test, and considered *p* values less than 0.05 to indicate statistical significance. All statistical analyses were performed using python (version 3.9.1) and various machine learning libraries such as scikit‐learn.

## AUTHOR CONTRIBUTIONS

N. L., X. Z., C. H., H. S., and X. M. were involved in the study conception and design. N. L. and X. Z. extracted the data and completed the data analysis. C. H. managed data acquisition and contributed to study design. Z. L., T. S., L. Z., and T. D. contributed to data acquisition. W. C., Y. W., Z. L., T. S., L. Z., C. Y., T. D., and H. Z. contributed to data interpretation. X. W. contributed to revising and polishing the language of the paper. All authors contributed to data interpretation, writing, and reviewing the manuscript. All authors had access to all the data in the study and had final responsibility for the decision to submit the research for publication. All authors have read and approved the final manuscript.

## CONFLICT OF INTEREST STATEMENT

The author Chengyuan He is a shareholder and member of the board of directors of Recovery Plus Inc., a company that is developing medical digital devices to help clinicians, Zhiwen Long is also employed by Recovery Plus Inc.. Recovery Plus Clinic is an out clinic attached to Recovery Plus Inc. As a private hospital, it does not have an ethics committee to conduct independent ethical review, but participates in this clinical trial as a sub‐center of clinical trial. Recovery Plus Inc. provide financial support to the curent study. Chenyuan He and Zhiwen Long from this company contributed to the study design, data collection, data analysis, data interpretation, and writing of the report. The author's involvement with the company could potentially bias their interpretation of the results presented here. However, the author has taken measures to minimize this potential bias, including conducting independent analyses and involving other researchers with no affiliation to the company in the study design and data analysis. The other authors have no conflicts of interest to declare.

## CODE AVAILABILITY

The software and source code are the property of Recovery Plus Inc. Any enquiry for access to these should be made directly to Recovery Plus Inc.

## ETHICS STATEMENT

The study was registered in ChiCTR with registration number ChiCTR2200063512. This study also obtained approval for conducting research at West China Hospital (approval number: 20221153), Sichuan University Huaxi Hospital Tibet Chengban Branch (approval number: 202273) and Yunnan Cancer Hospital (approval number: SLKYCS2021240). Informed consent statement was gained from all of the patients.

## Supporting information

Supporting Information

## Data Availability

After signing a data use agreement, deidentified data that do not violate confidentiality can be made available upon reasonable request from the date of publication. The agreement will specify that the deidentified data can only be used for research purposes, and not for product related work, and cannot be shared with a third party. Additionally, related documents can also be made available on reasonable request; please contact the corresponding author.
