# Supplementary material for: Development and validation of a point‐of‐care nursing mobile tool to guide the diagnosis of malnutrition in hospitalized adult patients: a multicenter, prospective cohort study
Source: MedComm (2020). 2024 Apr 10;5(4):e526. doi: 10.1002/mco2.526 (PMC11006711; doi:10.1002/mco2.526)
Supplement: Supplementary file 1 — Supporting Information [file MCO2-5-e526-s001.docx]

Development and validation of a point-of-care nursing mobile tool to guide the diagnosis of malnutrition in hospitalized adult patients: a multicenter, prospective cohort study

Nan Lin1*, Xueyan Zhou2*, Weichang Chen3*, Chengyuan He4*, Xiaoxuan Wang1, Yuhao Wei1, Zhiwen Long4, Tao Shen5, Lingyu Zhong6, Chan Yang7, Tingting Dai8, Hao Zhang9, Hubing Shi10†, Xuelei Ma1†

1 Department of Biotherapy, Cancer Center, West China Hospital, Sichuan University, Chengdu, China

2 Department of Biotherapy, State Key Laboratory of Biotherapy, Frontiers Science Center for Disease-related Molecular Network, West China Hospital, and Key Laboratory of Bio-Resource and Eco-Environment of Ministry of Education, College of Life Sciences, Sichuan University, Chengdu, Sichuan, China

3 State Key Laboratory of Oral Diseases, National Clinical Research Center for Oral Diseases, Sichuan University, Chengdu, China

4 Recovery Plus Clinic, Chengdu, China

5 Department of Colorectal Surgery, The Third Affiliated Hospital of Kunming Medical University/Yunnan Tumor Hospital, Kunming, China

6 Hospital of Chengdu Office of People’s Government of Tibetan, Chengdu, China

7 Division of Endocrinology and Metabolism, State Key Laboratory of Biotherapy, West China Hospital, Sichuan University, Chengdu, China

8 Department of Clinical Nutrition, West China Hospital, Sichuan University, Chengdu, China

9 Division of Pancreatic Surgery, Department of General Surgery, West China Hospital, Sichuan University, Chengdu, China

10 Laboratory of Integrative Medicine, Clinical Research Center for Breast, State Key Laboratory of Biotherapy, West China Hospital, Sichuan University and Collaborative Innovation Center, Chengdu, Sichuan, China

Nan Lin, Xueyan Zhou, Weichang Chen, Chengyuan He contributed equally and should be listed as co-first authors.

† Corresponding author:

Xuelei Ma

West China Hospital, Guoxue Lane 37, Chengdu, China.

Electronic address: drmaxuelei@gmail.com; [maxuelei0726@wchscu.cn](mailto:maxuelei0726@wchscu.cn)

Hubing Shi

West China Hospital, Guoxue Lane 37, Chengdu, China.

Email address: shihb@scu.edu.cn

**Chapter 1**

**Background and Purpose**

**1.1 Objectives**

This study is a prospective and observational trial with the following objectives:

- To find out a good standard for identifying malnutrition.
- To explore facial regions suitable for building nutrition recognition models.
- To develop and evaluate the performance of the Malnutrition Screening Eye (MSE).
- To test the MSE system in real-world application

**1.2 Rationale of the Study**

The study is conducted for the following reasons:

- Malnutrition is a global issue, with 828 million people are affected. It has a huge socio-economic impact on countries, and the diagnosis and treatment of malnutrition is a major challenge.
- Certain features of patients, such as facial appearance, can assist malnutritional diagnosis; however, recording and applying these features for clinical practice in the real world are still challenging.
- To develop a reliable and effective tool for clinical practical for screening the malnutrition for inpatients in real-world settings, we wanted to develop a smartphone-based system called “MSE” to analyze phenotypic features using machine learning.
- We also performed a prospective and observational trial to investigate whether the AIS system can be applied in real-world settings.

**1.3 Summary of Study Protocol**

Study Sites:

In this study, we retrieved patients’ data from four site: West China Hospital (Chengdu, China), Sichuan University Huaxi Hospital Tibet Chengban Branch (Chengdu, China), Yunnan Cancer Hospital (Yunnan, China), Recovery Plus Clinic (Chengdu, China).

Primary Outcome:

The area under the receiver operating curve (AUC) for MSE to identify visual impairment.

Secondary Outcomes:

The accuracy, sensitivity, specificity, and reliability of the MSE system.

**Chapter 2**

**Patient Recruitment and Enrollment**

**2.1 Participant Recruitment**

Patients will be retrieved patients’ data from four site: West China Hospital (Chengdu, China), Sichuan University Huaxi Hospital Tibet Chengban Branch (Chengdu, China), Yunnan Cancer Hospital (Yunnan, China), Recovery Plus Clinic (Chengdu, China).

**2.2 Informed Consent and Enrollment**

Written informed consent will be obtained from the patients during the doctors taking facial photos and explain the questionnaire results to them.

**2.3 Eligibility Criteria**

- Participants who are 18 years of age or older and possess the cognitive ability to complete a questionnaire.
- Participants able to be gained the informed consent.

**2.4 Exclusion Criteria**

- The patient’s condition is so dire to filled in the questionnaire.
- Patient has obvious skin lesions on the face and face recognition cannot be performed.

**2.5 Nutritional Assess Procedures and Photo Obtain**

The nutritional assess was based on a mini program called R+ Dietitian. R+ Dietitian was a mobile-phone based mini program for nutritional risk screening and dietary assessment. In the previous study, we have compared R+ Dietitian with experienced dietitians, which showed high accuracy, sensitivity, and specificity. The basic function of this mini program was collecting of basic information of patients, and screening their nutritional risk. After the nutritional assessment, participants can choose whether to take a picture to gain their face data for analysis. The information needed to be collected were showed in the following figure.

In the 5 included hospitals, we licensed it to doctors or nurses in relative specialties, like oncology department, surgery departments, and endocrine department to use the application as daily nutritional assessment tool. The included participants can be divided into cancer inpatients, other inpatients and normal control, based on their disease situation. First, all patients’ informed consent were gained. Second, with the doctors’ or the nurses’ instruction, the patients will finish the questionnaires in the mini program. Next, there will be an optional choice for them in the mini program, to take a photo or not. After completing the participants’ daily nutritional assessment, doctors or nurses would instruct the patient to take facial photos and explain the questionnaire results to them. All doctors and nurses were not specialized dietitian.

**Chapter 3**

**Statistical Analyses**

**3.1 Feasibility Analysis**

We conducted a Pearson correlation analysis to determine the correlation between the scores obtained from the two systems. Pearson correlation analysis is a commonly used statistical method for analyzing the linear relationship between two variables. It measures the correlation between two variables by calculating their covariance. The greater the value of covariance, the stronger the correlation between the two variables. The calculation formula is as follows, where $x_{i}$ and$y_{i}$ represent the $i$-th observed value of the two variables, $\bar{x}$ and $\bar{y}$ represent the mean values of the two variables, and $n$ represents the sample size:

$$r=\frac{\sum_{i=1}^{n} \sum(x_{i}-\bar{x})(y_{i}-\bar{y})}{\sqrt{\sum_{i=1}^{n} {(x_{i}-\bar{x})}^{2}\sum_{i=1}^{n} {(y_{i}-\bar{y})}^{2}}}$$

**3.2 Required Models’ Algorithm Principle**

We used Adaboost, Extratree, Random Forest (RF), Xgboost, and KNN models to compare the classification performance of full-face features excluding eyes and ocular features in the PG-SGA SF system for facial assessment section.

**3.2.1 AdaBoost**

The basic principle of the Adaboost algorithm is to initialize the weights of the training set and train each weak classifier. Based on the classification error of each weak classifier, the weights of the samples are updated, reducing the weight of correctly classified samples and increasing the weight of misclassified samples. In this way, misclassified samples will receive more attention in subsequent iterations. Following the same method sequentially, the iteration continues until all weak classifiers are integrated to form a strong classifier. The core idea of the algorithm is to combine several homogeneous classifiers into a strong classifier through linear weighting, so it is mainly used to calculate the weight of each weak classifier. Specifically, for a given weak classifier, the initial data set is used for fitting, and then the weight of the classifier is obtained based on the fitting result and the sample weights of the data set are adjusted. By this method, the Adaboost algorithm can focus on handling difficult-to-classify samples in each iteration, thereby improving the classification accuracy. At the same time, by continuously updating the weights of the samples through iteration, the algorithm can gradually improve the accuracy of each weak classifier, and finally build a strong classifier with high accuracy. This mechanism enables the Adaboost algorithm to focus on solving difficult-to-classify samples. The specific Adaboost calculation method is as follows:

1. Initialization of training sample weights: In the absence of any prior knowledge, the weights of each of the N training samples are initialized as 1/N.
2. Computing classifier error a: The weight of each classifier is calculated based on its accuracy in each round of classification. The higher the accuracy, the greater the weight of the classifier. The following formula shows the computation of the error rate a in the t-th iteration for sample $\varepsilon_{t}$, where $h_{t}(x_{i})$ represents the classification category of $x_{i}$ after $t$ iterations, $y_{i}$ represents the true classification label of sample $x_{i}$, and $D_{i}^{t}$ represents the weight of sample $i$ in the $t$ iteration:

$$\varepsilon_{t}=\sum_{i=1}^{N} D_{i}^{t}\left[ h_{t}\left( x_{i} \right)\neq y_{i} \right]$$

3. Calculate the weight of the classifier $\alpha$: Based on the classification error obtained in step (2), determine the proportion coefficient of the weak classifier in the final strong classifier:

$$\alpha=\frac{1}{2}\log\frac{1-\varepsilon_{t}}{\varepsilon_{t}}$$

4. Update training sample weights: Update the weights of the training samples based on the classification results obtained in step (2) and the classifier weights obtained in step (3):

$$D_{i}^{t+1}=D_{i}^{t}\cdot exp(a^{t}\cdot[h_{t}\left( x_{i} \right)\neq y_{i}])$$

5. The process of outputting a strong classifier involves linearly combining the weights and classification accuracy of individual classifiers.

**3.2.2 Extratree**

ExtraTrees is an ensemble learning algorithm based on decision trees, which is further improved based on random forests. Unlike random forests, ExtraTrees not only randomly selects features when generating each decision tree, but also randomly selects splitting points. This randomness makes ExtraTrees more diverse and less variable, thus more robust and effective. The specific ExtraTrees calculation method is as follows:

1. For a dataset with m features, randomly select $k$ features for splitting, where $k$ = $\sqrt{m}$. For each feature $i$, its split point is denoted as $t_{i}$, $C$ is the number of classes, and $p_{c}$, is the proportion of samples in the $c$-th class. Its Gini impurity is:

$$Gini=1-\sum_{c=1}^{C} P_{C}^{2}$$

2. For each splitting point $t_{i}$, its information gain can be calculated.：

$$IG={Gini}_{before}- {Gini}_{after}$$

3. The generated multiple decision trees are combined into an ExtraTrees model for classification or regression tasks.

**3.2.3 Random Forest**

Random Forest is an ensemble learning method based on decision tree construction, aimed at reducing the risk of overfitting and improving the accuracy of the model. Its basic idea is to simultaneously train multiple decision trees, and then combine their results to form a more stable prediction result. The specific Random Forest calculation method is as follows:

1. For each training set, a decision tree algorithm is used to generate a decision tree. Here, $T_{i}$ represents the $i$-th decision tree and $D_{i}$ represents the $i$-th randomly sampled training set:

$$T_{i}=DecisionTree(D_{i})$$

2. For each decision tree node, a certain number of features are randomly selected for feature selection, where $m$ represents the number of features selected in this feature selection and $p$ represents the total number of features:

$$m=\sqrt{p}$$

3. For new data, predictions are made using multiple decision trees, and the final prediction is obtained by voting or taking a weighted average of the predictions of each tree. Here, y represents the final prediction result, n represents the number of decision trees, and $f_{i}(x)$ represents the prediction result of the $i$-th decision tree:

$$y=\frac{1}{n}\sum_{i=1}^{n} f_{i}(x)$$

**3.2.4 XGBoost**

XGBoost is an ensemble learning algorithm used to solve classification and regression problems. It combines gradient boosting decision trees and regularization techniques to improve the performance and robustness of the model. The specific loss function and objective function are set as follows: where $l$ is the loss function, $y_{i}$ is the true label of the $i$-th sample, $\hat{y_{i}}$ is the predicted label of the $i$-th sample, $K$ is the number of trees, $f_{k}$ is the $k$-th tree, and ${\Omega(f}_{k})$ is the regularization term which helps to prevent overfitting. L2 regularization is adopted:

l$oss= \sum_{i=1}^{n} l\left( y_{i} , \hat{y_{i}} \right)+\sum_{k=1}^{K} {\Omega(f}_{k})$

**3.2.5 k-NN**

The k-NN algorithm is an instance-based supervised learning algorithm. The algorithm is based on a simple idea that the label of a sample is determined by the labels of the k-nearest neighbors to the target sample. Specifically, the k-NN algorithm determines the nearest k neighbors by calculating the distance or similarity between the target sample and each training sample, and classifies or regresses based on the labels of these neighbors. The specific calculation method of the k-NN algorithm is as follows:

(1) Input the training dataset and target sample.

(2) Calculate the distance or similarity between the target sample and each sample in the training set, $x$ and $y$ are two vectors, and $n$ is the dimension of the vectors.：

$$d\left( x,y \right)=\sqrt{\sum_{i=1}^{n} {(x_{i}-y_{i})}^{2}}$$

(3) Find the $k$ nearest neighbors to the target sample based on the distance or similarity.

(4) Make a classification prediction based on the labels of the $k$ neighbors, $N_{k}(i_{0})$ is the set of k-nearest neighbors of the target sample $i_{0}$, $y_{i}$ is the label of the training sample $i$, and ${[y}_{i}=c_{j}]$ is an indicator function that takes the value of 1 if $y_{i}=c_{j}$ and 0 otherwise.：

$$y=arg{max}_{c_{j}}\sum_{i\in N_{k}(i_{0})} {[y}_{i}=c_{j}]$$

**3.3 Statistical Analysis for Study Endpoints**

**3.3.1 Accuracy Endpoints**

To evaluate the classification performance of various models, we utilized six metrics, including accuracy, sensitivity, specificity, positive predictive value (PPV), negative predictive value (NPV), and area under the curve (AUC), and computed the 95% CI for each indicator. Bootstrap method was used to determine the 95% CI of the external verification cohort in 1000 sampling times.

Accuracy:

It is defined as the ratio of the number of correct predictions to the total number of all predictions.

Sensitivity:

Sensitivity is defined as the ratio of positive predictions to the total number of actual positive cases, also known as the true positive rate.

Specificity:

Specificity is defined as the ratio of negative predictions to the total number of actual negative cases, also known as the true negative rate.

Positive predictive value:

PPV is defined as the ratio of actual positive cases among the total predicted positive cases.

Negative prediction value:

NPV is defined as the ratio of actual negative cases among the total predicted negative cases.

Area under curve:

AUC is defined as the probability that a randomly chosen positive example is ranked higher than a randomly chosen negative example. A higher AUC indicates a better classification performance of a definite predictor.

**3.3.2 Comparative Endpoints**

In order to facilitate the comparison of receiver operating characteristic (ROC) curves obtained by different models under the same training cohort, we conducted DeLong test, Integrated Discrimination Improvement (IDI) test, and Net Reclassification Index (NRI) test, and considered *p*-values less than 0.05 to indicate statistical significance.

DeLong Test:

The DeLong test is a statistical test used to compare the performance of two correlated diagnostic tests in terms of their ability to correctly classify individuals into a binary outcome (such as disease vs. no disease). The DeLong test is a nonparametric test that compares the area under the receiver operating characteristic (ROC) curves of the two tests.

IDI Test:

IDI is a statistical measure that is used to evaluate the improvement in predictive accuracy of a model when a new predictor is added. IDI can be interpreted as the proportion of the variance in predicted probabilities explained by the addition of the new predictor, and is often reported as a percentage.

NRI Test:

NRI is a statistical measure used to assess the improvement in the accuracy of risk prediction models. Specifically, the NRI quantifies the proportion of individuals who are correctly reclassified into more accurate risk categories by a new prediction model compared to an old model. The NRI is calculated by summing the differences between the proportions of individuals who are correctly reclassified upward and downward by the new model compared to the old model. The resulting value ranges from -1 to 1, with higher values indicating better reclassification performance of the new model.

**Table S1: Pearson test for correlation analysis between NRS2002 and PG-SGA SF**

| **Group name** | **Related coefficient** | ***p* value** |
| --- | --- | --- |
| Cancer Inpatient Group | 0.575 | <0.001^a^ |
| Normal Control Group | 0.402 | <0.005^a^ |
| Other Inpatient Group | 0.515 | <0.001^a^ |
| All Inpatient Group | 0.433 | <0.001^a^ |
| Note: Data were related coefficient obtained after pearson-test. ^a^*p* <0.05 indicated significant difference between models in the test. | | | |

**Table S2: Results of different classification models to identify the nutritional status with different inpatient groups**

|  |  | **Ocular features** | | | | | |
| --- | --- | --- | --- | --- | --- | --- | --- |
|  |  | **Accuracy (95% CI)** | **Sensitivity (95% CI)** | **Specificity (95% CI)** | **PPV (95% CI)** | **NPV (95% CI)** | **AUC (95% CI)** |
| Cancer Inpatient Group | AdaBoost | 0.708(0.611-0.806) | 0.826(0.780-0.872) | 0.500(0.317-0.683) | 0.745(0.650-0.840) | 0.619(0.509-0.729) | 0.726(0.664-0.789) |
|  |  | 0.667(0.498-0.835) | 0.750(0.691-0.809) | 0.536(0.482-0.590) | 0.717(0.661-0.773) | 0.577(0.423-0.730) | 0.778(0.735-0.820) |
|  |  | 0.694(0.590-0.799) | 0.812(0.754-0.871) | 0.458(0.383-0.533) | 0.750(0.638-0.862) | 0.550(0.460-0.640) | 0.719(0.646-0.791) |
|  | Extratree | 0.583(0.474-0.693) | 0.609(0.522-0.695) | 0.538(0.406-0.671) | 0.700(0.648-0.752) | 0.438(0.348-0.527) | 0.725(0.660-0.789) |
|  |  | 0.625(0.552-0.698) | 0.769(0.695-0.844) | 0.455(0.311-0.598) | 0.625(0.526-0.724) | 0.625(0.458-0.792) | 0.736(0.692-0.779) |
|  |  | 0.625(0.569-0.681) | 0.651(0.581-0.722) | 0.586(0.446-0.726) | 0.700(0.616-0.784) | 0.531(0.397-0.666) | 0.735(0.683-0.787) |
|  | Random  Forest | 0.625(0.500-0.750) | 0.868(0.768-0.969) | 0.353(0.271-0.435) | 0.600(0.555-0.645) | 0.706(0.573-0.839) | 0.724(0.658-0.789) |
|  |  | 0.625(0.552-0.698) | 0.725(0.635-0.815) | 0.500(0.332-0.668) | 0.644(0.554-0.735) | 0.593(0.539-0.646) | 0.709(0.634-0.784) |
|  |  | 0.583(0.454-0.713) | 0.667(0.563-0.770) | 0.444(0.331-0.557) | 0.667(0.572-0.762) | 0.444(0.360-0.529) | 0.663(0.594-0.733) |
|  | XGBoost | 0.583(0.519-0.647) | 0.673(0.623-0.724) | 0.391(0.334-0.448) | 0.702(0.643-0.762) | 0.360(0.306-0.414) | 0.776(0.732-0.821) |
|  |  | 0.639(0.558-0.720) | 0.690(0.640-0.741) | 0.567(0.394-0.740) | 0.690(0.589-0.792) | 0.567(0.400-0.733) | 0.690(0.629-0.752) |
|  |  | 0.597(0.509-0.686) | 0.647(0.543-0.751) | 0.476(0.326-0.627) | 0.750(0.688-0.812) | 0.357(0.206-0.508) | 0.715(0.674-0.755) |
|  | k-NN | 0.681(0.586-0.775) | 0.820(0.759-0.881) | 0.364(0.251-0.476) | 0.745(0.691-0.799) | 0.471(0.405-0.536) | 0.723(0.663-0.783) |
|  |  | 0.625(0.464-0.786) | 0.744(0.705-0.782) | 0.485(0.400-0.570) | 0.630(0.576-0.685) | 0.615(0.427-0.804) | 0.679(0.634-0.723) |
|  |  | 0.694(0.601-0.788) | 0.891(0.824-0.959) | 0.346(0.182-0.510) | 0.707(0.591-0.823) | 0.643(0.505-0.781) | 0.707(0.666-0.748) |
| Other Inpatient Group | AdaBoost | 0.767(0.654-0.879) | 0.872(0.782-0.962) | 0.571(0.411-0.732) | 0.791(0.726-0.855) | 0.706(0.574-0.838) | 0.842(0.781-0.904) |
|  |  | 0.733(0.583-0.884) | 0.814(0.778-0.850) | 0.529(0.416-0.642) | 0.814(0.702-0.926) | 0.529(0.474-0.585) | 0.845(0.799-0.891) |
|  |  | 0.767(0.697-0.836) | 0.824(0.768-0.879) | 0.692(0.569-0.815) | 0.778(0.677-0.878) | 0.750(0.627-0.873) | 0.794(0.733-0.854) |
|  | Extratree | 0.767(0.639-0.894) | 0.800(0.703-0.897) | 0.700(0.643-0.757) | 0.842(0.733-0.951) | 0.636(0.557-0.716) | 0.837(0.798-0.876) |
|  |  | 0.817(0.741-0.892) | 0.825(0.747-0.903) | 0.800(0.750-0.850) | 0.892(0.852-0.932) | 0.696(0.617-0.774) | 0.846(0.777-0.914) |
|  |  | 0.667(0.519-0.814) | 0.800(0.709-0.891) | 0.533(0.360-0.707) | 0.632(0.523-0.741) | 0.727(0.587-0.868) | 0.702(0.651-0.752) |
|  | Random  Forest | 0.700(0.550-0.850) | 0.805(0.751-0.859) | 0.474(0.402-0.546) | 0.767(0.664-0.871) | 0.529(0.403-0.656) | 0.791(0.746-0.836) |
|  |  | 0.683(0.628-0.739) | 0.762(0.719-0.805) | 0.500(0.409-0.591) | 0.780(0.670-0.891) | 0.474(0.285-0.663) | 0.777(0.738-0.816) |
|  |  | 0.700(0.575-0.825) | 0.732(0.680-0.784) | 0.632(0.524-0.739) | 0.811(0.746-0.875) | 0.522(0.339-0.704) | 0.766(0.709-0.824) |
|  | XGBoost | 0.700(0.536-0.864) | 0.839(0.739-0.938) | 0.552(0.438-0.665) | 0.667(0.607-0.727) | 0.762(0.583-0.941) | 0.770(0.722-0.818) |
|  |  | 0.700(0.644-0.756) | 0.825(0.780-0.870) | 0.450(0.390-0.510) | 0.750(0.660-0.840) | 0.562(0.410-0.715) | 0.784(0.731-0.837) |
|  |  | 0.600(0.500-0.700) | 0.659(0.550-0.767) | 0.474(0.304-0.643) | 0.730(0.648-0.811) | 0.391(0.305-0.478) | 0.716(0.668-0.763) |
|  | k-NN | 0.783(0.622-0.944) | 0.868(0.780-0.957) | 0.636(0.534-0.738) | 0.805(0.696-0.914) | 0.737(0.616-0.857) | 0.819(0.779-0.859) |
|  |  | 0.750(0.589-0.911) | 0.850(0.760-0.940) | 0.550(0.470-0.630) | 0.791(0.724-0.857) | 0.647(0.504-0.791) | 0.807(0.753-0.862) |
|  |  | 0.683(0.526-0.840) | 0.879(0.828-0.930) | 0.444(0.301-0.588) | 0.659(0.598-0.720) | 0.750(0.587-0.913) | 0.746(0.678-0.815) |
| All Inpatient Group | AdaBoost | 0.655(0.523-0.787) | 0.797(0.695-0.899) | 0.460(0.366-0.554) | 0.671(0.555-0.787) | 0.622(0.562-0.681) | 0.753(0.705-0.802) |
|  |  | 0.672(0.609-0.735) | 0.795(0.757-0.833) | 0.389(0.319-0.459) | 0.750(0.700-0.800) | 0.452(0.306-0.597) | 0.740(0.690-0.791) |
|  |  | 0.655(0.499-0.812) | 0.829(0.752-0.906) | 0.349(0.172-0.525) | 0.692(0.643-0.741) | 0.536(0.362-0.710) | 0.747(0.695-0.798) |
|  | Extratree | 0.647(0.525-0.769) | 0.811(0.711-0.911) | 0.378(0.231-0.524) | 0.682(0.565-0.798) | 0.548(0.473-0.623) | 0.754(0.694-0.814) |
|  |  | 0.731(0.658-0.804) | 0.840(0.766-0.914) | 0.545(0.490-0.601) | 0.759(0.659-0.859) | 0.667(0.497-0.836) | 0.792(0.750-0.834) |
|  |  | 0.706(0.538-0.874) | 0.913(0.882-0.944) | 0.420(0.355-0.485) | 0.685(0.637-0.732) | 0.778(0.623-0.932) | 0.771(0.726-0.815) |
|  | Random  Forest | 0.739(0.641-0.838) | 0.813(0.780-0.846) | 0.614(0.439-0.788) | 0.782(0.693-0.871) | 0.659(0.474-0.843) | 0.790(0.723-0.858) |
|  |  | 0.723(0.584-0.861) | 0.886(0.855-0.916) | 0.490(0.307-0.673) | 0.713(0.659-0.767) | 0.750(0.568-0.932) | 0.763(0.725-0.801) |
|  |  | 0.689(0.573-0.805) | 0.877(0.809-0.945) | 0.463(0.363-0.562) | 0.663(0.574-0.752) | 0.758(0.569-0.946) | 0.731(0.664-0.798) |
|  | XGBoost | 0.739(0.647-0.832) | 0.833(0.730-0.937) | 0.561(0.435-0.687) | 0.783(0.725-0.841) | 0.639(0.572-0.706) | 0.752(0.688-0.816) |
|  |  | 0.782(0.665-0.898) | 0.873(0.792-0.955) | 0.600(0.462-0.738) | 0.812(0.697-0.926) | 0.706(0.644-0.767) | 0.785(0.711-0.858) |
|  |  | 0.689(0.606-0.772) | 0.806(0.728-0.883) | 0.511(0.343-0.678) | 0.716(0.636-0.796) | 0.632(0.451-0.812) | 0.741(0.670-0.812) |
|  | k-NN | 0.706(0.612-0.799) | 0.789(0.684-0.895) | 0.558(0.384-0.733) | 0.759(0.692-0.827) | 0.600(0.472-0.728) | 0.825(0.758-0.892) |
|  |  | 0.697(0.551-0.844) | 0.928(0.886-0.969) | 0.380(0.208-0.552) | 0.674(0.626-0.721) | 0.792(0.623-0.961) | 0.788(0.727-0.850) |
|  |  | 0.639(0.559-0.719) | 0.765(0.725-0.805) | 0.324(0.218-0.429) | 0.739(0.622-0.856) | 0.355(0.192-0.518) | 0.733(0.666-0.799) |
|  |  | **Full-face features excluding eyes** | | | | | |
|  |  | **Accuracy (95% CI)** | **Sensitivity (95% CI)** | **Specificity (95% CI)** | **PPV (95% CI)** | **NPV (95% CI)** | **AUC (95% CI)** |
| Cancer Inpatient Group | AdaBoost | 0.611(0.442-0.781) | 0.857(0.805-0.909) | 0.267(0.147-0.386) | 0.621(0.579-0.662) | 0.571(0.512-0.631) | 0.672(0.622-0.721) |
|  |  | 0.611(0.453-0.770) | 0.732(0.669-0.794) | 0.452(0.279-0.625) | 0.638(0.582-0.695) | 0.560(0.391-0.729) | 0.678(0.632-0.723) |
|  |  | 0.681(0.628-0.733) | 0.860(0.819-0.902) | 0.414(0.332-0.496) | 0.685(0.594-0.776) | 0.667(0.548-0.785) | 0.652(0.593-0.711) |
|  | Extratree | 0.583(0.522-0.644) | 0.681(0.627-0.734) | 0.400(0.282-0.518) | 0.681(0.586-0.776) | 0.400(0.252-0.548) | 0.676(0.627-0.724) |
|  |  | 0.625(0.521-0.729) | 0.619(0.580-0.658) | 0.633(0.464-0.803) | 0.703(0.608-0.798) | 0.543(0.475-0.610) | 0.655(0.582-0.729) |
|  |  | 0.597(0.511-0.684) | 0.902(0.853-0.952) | 0.194(0.066-0.321) | 0.597(0.533-0.661) | 0.600(0.485-0.715) | 0.632(0.569-0.696) |
|  | Random  Forest | 0.528(0.432-0.623) | 0.582(0.509-0.655) | 0.353(0.267-0.439) | 0.744(0.700-0.789) | 0.207(0.063-0.350) | 0.614(0.539-0.688) |
|  |  | 0.542(0.480-0.603) | 0.630(0.522-0.739) | 0.385(0.253-0.516) | 0.644(0.569-0.720) | 0.370(0.236-0.505) | 0.615(0.558-0.673) |
|  |  | 0.583(0.476-0.691) | 0.610(0.574-0.645) | 0.548(0.385-0.711) | 0.641(0.562-0.720) | 0.515(0.419-0.611) | 0.644(0.577-0.712) |
|  | XGBoost | 0.611(0.454-0.768) | 0.667(0.563-0.771) | 0.533(0.474-0.593) | 0.667(0.607-0.726) | 0.533(0.358-0.709) | 0.696(0.630-0.762) |
|  |  | 0.597(0.441-0.754) | 0.682(0.579-0.784) | 0.464(0.306-0.623) | 0.667(0.589-0.745) | 0.481(0.397-0.566) | 0.685(0.629-0.740) |
|  |  | 0.556(0.387-0.724) | 0.641(0.592-0.690) | 0.455(0.287-0.622) | 0.581(0.524-0.639) | 0.517(0.438-0.596) | 0.603(0.537-0.669) |
|  | k-NN | 0.611(0.516-0.706) | 0.698(0.599-0.798) | 0.368(0.253-0.483) | 0.755(0.693-0.817) | 0.304(0.176-0.433) | 0.674(0.603-0.745) |
|  |  | 0.597(0.451-0.743) | 0.714(0.623-0.805) | 0.348(0.288-0.408) | 0.700(0.587-0.813) | 0.364(0.282-0.445) | 0.659(0.609-0.708) |
|  |  | 0.639(0.530-0.748) | 0.769(0.669-0.870) | 0.485(0.347-0.622) | 0.638(0.581-0.696) | 0.640(0.559-0.721) | 0.683(0.616-0.750) |
| Other Inpatient Group | AdaBoost | 0.683(0.604-0.763) | 0.694(0.661-0.728) | 0.667(0.578-0.756) | 0.758(0.651-0.864) | 0.593(0.463-0.722) | 0.663(0.617-0.708) |
|  |  | 0.617(0.489-0.745) | 0.684(0.584-0.784) | 0.500(0.428-0.572) | 0.703(0.642-0.763) | 0.478(0.389-0.567) | 0.607(0.559-0.655) |
|  |  | 0.683(0.615-0.752) | 0.810(0.753-0.866) | 0.389(0.312-0.466) | 0.756(0.681-0.830) | 0.467(0.369-0.564) | 0.669(0.624-0.715) |
|  | Extratree | 0.700(0.593-0.807) | 0.868(0.804-0.933) | 0.409(0.317-0.501) | 0.717(0.616-0.819) | 0.643(0.498-0.788) | 0.693(0.637-0.750) |
|  |  | 0.717(0.556-0.877) | 0.756(0.709-0.803) | 0.632(0.546-0.717) | 0.816(0.701-0.931) | 0.545(0.381-0.710) | 0.802(0.756-0.849) |
|  |  | 0.683(0.551-0.815) | 0.757(0.665-0.849) | 0.565(0.477-0.653) | 0.737(0.625-0.849) | 0.591(0.427-0.755) | 0.771(0.703-0.838) |
|  | Random  Forest | 0.633(0.541-0.725) | 0.758(0.650-0.865) | 0.481(0.321-0.642) | 0.641(0.541-0.741) | 0.619(0.470-0.769) | 0.701(0.643-0.759) |
|  |  | 0.700(0.580-0.820) | 0.810(0.704-0.916) | 0.444(0.310-0.579) | 0.773(0.719-0.826) | 0.500(0.343-0.657) | 0.737(0.702-0.773) |
|  |  | 0.567(0.430-0.704) | 0.645(0.539-0.751) | 0.483(0.345-0.620) | 0.571(0.518-0.625) | 0.560(0.425-0.695) | 0.623(0.572-0.673) |
|  | XGBoost | 0.667(0.615-0.718) | 0.750(0.671-0.829) | 0.500(0.328-0.672) | 0.750(0.690-0.810) | 0.500(0.336-0.664) | 0.751(0.698-0.805) |
|  |  | 0.633(0.546-0.720) | 0.641(0.569-0.713) | 0.619(0.465-0.773) | 0.758(0.656-0.859) | 0.481(0.423-0.540) | 0.707(0.643-0.771) |
|  |  | 0.583(0.482-0.685) | 0.659(0.615-0.702) | 0.421(0.354-0.488) | 0.711(0.592-0.829) | 0.364(0.251-0.477) | 0.662(0.597-0.726) |
|  | k-NN | 0.667(0.508-0.825) | 0.714(0.620-0.808) | 0.556(0.476-0.635) | 0.789(0.728-0.851) | 0.455(0.269-0.640) | 0.705(0.646-0.763) |
|  |  | 0.583(0.531-0.636) | 0.800(0.721-0.879) | 0.280(0.097-0.463) | 0.609(0.548-0.669) | 0.500(0.434-0.566) | 0.644(0.583-0.705) |
|  |  | 0.733(0.617-0.849) | 0.868(0.816-0.921) | 0.500(0.412-0.588) | 0.750(0.658-0.842) | 0.688(0.531-0.844) | 0.756(0.720-0.793) |
| All Inpatient Group | AdaBoost | 0.672(0.509-0.836) | 0.882(0.817-0.947) | 0.302(0.138-0.467) | 0.691(0.604-0.778) | 0.591(0.500-0.682) | 0.698(0.643-0.753) |
|  |  | 0.639(0.541-0.736) | 0.912(0.881-0.944) | 0.077(0.014-0.140) | 0.670(0.600-0.740) | 0.300(0.125-0.475) | 0.680(0.635-0.725) |
|  |  | 0.630(0.461-0.800) | 0.890(0.828-0.952) | 0.217(0.121-0.314) | 0.644(0.563-0.725) | 0.556(0.384-0.727) | 0.662(0.622-0.702) |
|  | Extratree | 0.664(0.527-0.801) | 0.936(0.905-0.967) | 0.146(0.029-0.264) | 0.676(0.627-0.725) | 0.545(0.450-0.641) | 0.726(0.679-0.774) |
|  |  | 0.639(0.508-0.770) | 0.946(0.911-0.980) | 0.133(0.011-0.256) | 0.642(0.548-0.737) | 0.600(0.479-0.721) | 0.701(0.640-0.761) |
|  |  | 0.605(0.464-0.747) | 0.919(0.883-0.955) | 0.099(0.023-0.175) | 0.624(0.528-0.720) | 0.400(0.227-0.573) | 0.636(0.587-0.685) |
|  | Random  Forest | 0.681(0.518-0.844) | 0.868(0.821-0.916) | 0.349(0.252-0.446) | 0.702(0.629-0.775) | 0.600(0.445-0.755) | 0.695(0.657-0.733) |
|  |  | 0.605(0.467-0.743) | 0.775(0.702-0.847) | 0.354(0.214-0.494) | 0.640(0.567-0.712) | 0.515(0.370-0.660) | 0.617(0.555-0.678) |
|  |  | 0.538(0.421-0.655) | 0.776(0.674-0.878) | 0.231(0.075-0.386) | 0.565(0.497-0.634) | 0.444(0.385-0.504) | 0.598(0.547-0.649) |
|  | XGBoost | 0.664(0.598-0.729) | 0.759(0.712-0.805) | 0.406(0.223-0.589) | 0.776(0.736-0.817) | 0.382(0.221-0.543) | 0.713(0.640-0.786) |
|  |  | 0.672(0.605-0.740) | 0.805(0.715-0.895) | 0.378(0.237-0.520) | 0.742(0.673-0.810) | 0.467(0.282-0.652) | 0.693(0.656-0.729) |
|  |  | 0.647(0.483-0.812) | 0.740(0.675-0.805) | 0.476(0.324-0.628) | 0.722(0.660-0.783) | 0.500(0.432-0.568) | 0.647(0.579-0.715) |
|  | k-NN | 0.630(0.498-0.763) | 0.778(0.696-0.859) | 0.404(0.270-0.538) | 0.667(0.587-0.746) | 0.543(0.444-0.642) | 0.711(0.674-0.749) |
|  |  | 0.597(0.448-0.745) | 0.754(0.701-0.807) | 0.380(0.231-0.529) | 0.627(0.570-0.683) | 0.528(0.471-0.584) | 0.668(0.608-0.728) |
|  |  | 0.647(0.498-0.796) | 0.747(0.656-0.837) | 0.477(0.382-0.573) | 0.709(0.639-0.779) | 0.525(0.372-0.678) | 0.709(0.660-0.757) |

Note: PPV = Positive Predictive Value. NPV = Negative Predictive Value. CI = Confidence Interval. ROC = Receiver Operating Characteristic. RF = Random Forest. AUC = Area Under roc Curve.

**Table S3: Performance of face-eye weighted model to identify the nutritional status with different inpatient groups**

|  |  | **Accuracy(95% CI)** | **Sensitivity(95% CI)** | **Specificity(95% CI)** | **PPV(95% CI)** | **NPV(95% CI)** | **AUC (95% CI)** |
| --- | --- | --- | --- | --- | --- | --- | --- |
| Cancer Inpatient Group | Training cohort | 0.866  (0.798-0.933) | 0.857  (0.832-0.882) | 0.878  (0.789-0.966) | 0.909  (0.861-0.957) | 0.811  (0.712-0.911) | 0.940  (0.911-0.968) |
|  | Cross validation cohort | 0.840  (0.757-0.924) | 0.936  (0.909-0.962) | 0.659  (0.586-0.731) | 0.839  (0.743-0.935) | 0.844  (0.749-0.938) | 0.886  (0.843-0.930) |
|  | External verification cohort | 0.807  (0.727-0.887) | 0.896  (0.864-0.927) | 0.692  (0.613-0.772) | 0.789  (0.748-0.831) | 0.837  (0.748-0.927) | 0.860  (0.817-0.904) |
| Other Inpatient Group | Training cohort | 0.875  (0.814-0.936) | 0.894  (0.831-0.956) | 0.840  (0.781-0.899) | 0.913  (0.858-0.968) | 0.808  (0.762-0.853) | 0.940  (0.912-0.967) |
|  | Cross validation cohort | 0.792  (0.695-0.889) | 0.814  (0.736-0.892) | 0.759  (0.668-0.849) | 0.833  (0.756-0.911) | 0.733  (0.693-0.774) | 0.834  (0.764-0.904) |
|  | External verification cohort | 0.778  (0.642-0.913) | 0.886  (0.851-0.922) | 0.607  (0.520-0.694) | 0.780  (0.672-0.888) | 0.773  (0.696-0.850) | 0.843  (0.796-0.889) |
| All Inpatient Group | Training cohort | 0.850  (0.791-0.909) | 0.825  (0.777-0.873) | 0.900  (0.831-0.969) | 0.943  (0.917-0.968) | 0.720  (0.626-0.814) | 0.929  (0.893-0.965) |
|  | Cross validation cohort | 0.833  (0.727-0.940) | 0.971  (0.953-0.989) | 0.654  (0.597-0.711) | 0.786  (0.703-0.868) | 0.944  (0.91-0.978) | 0.927  (0.899-0.955) |
|  | External verification cohort | 0.800  (0.691-0.909) | 0.895  (0.832-0.957) | 0.636  (0.502-0.771) | 0.810  (0.730-0.889) | 0.778  (0.699-0.856) | 0.887  (0.829-0.944) |

Note: PPV = Positive Predictive Value. NPV = Negative Predictive Value. CI = Confidence Interval. ROC = Receiver Operating Characteristic. RF = Random Forest. AUC = Area Under roc Curve.


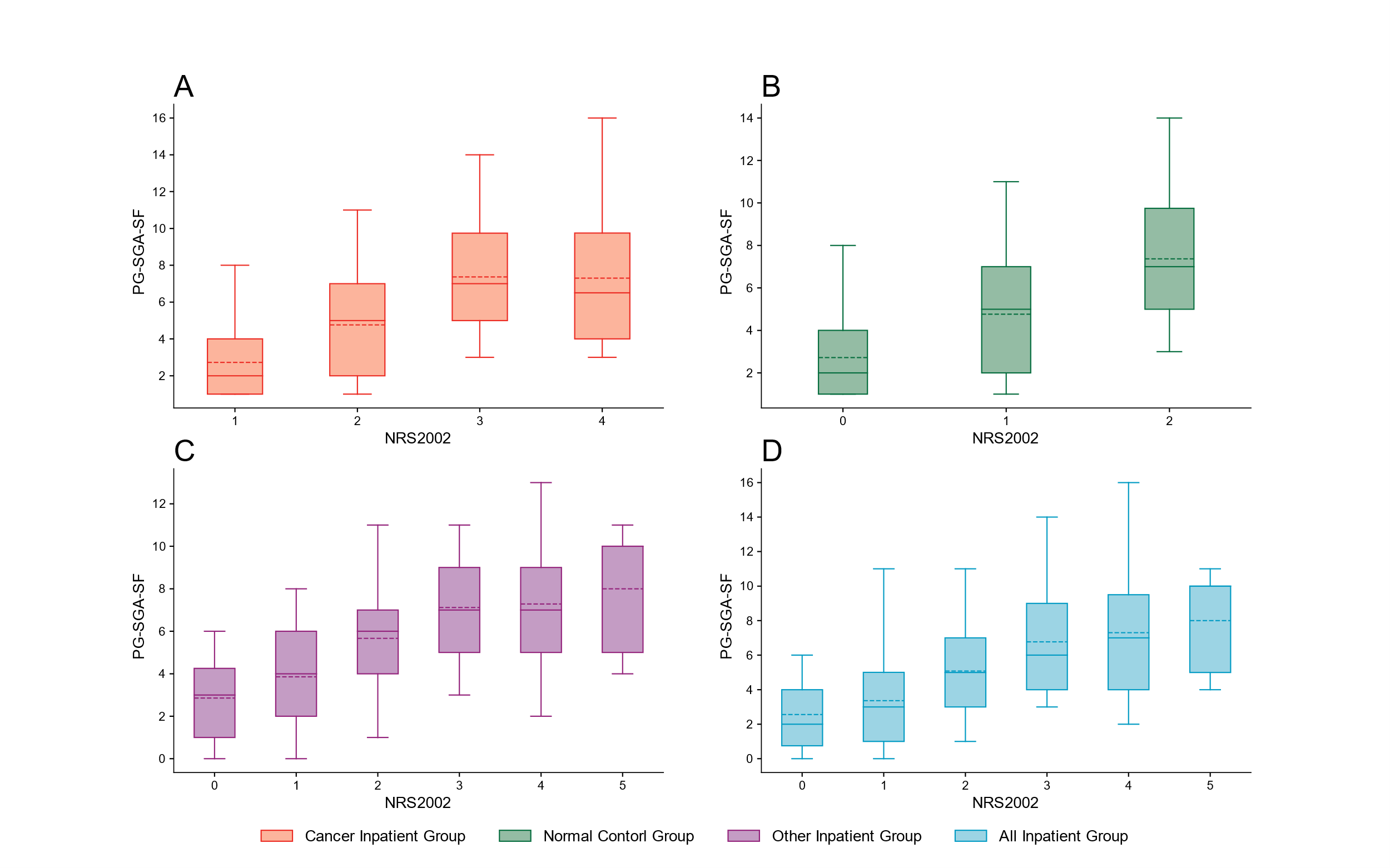


**Figure S1:Location and dispersion of NRS2002 and PG-SGA SF data for inpatient groups**

Box plots obtained from NRS2002 and PG-SGA SF for the four inclusion groups of Cancer Inpatient Group(A), Normal Control Group(B), Other Inpatient Group(C) and All Inpatient Group(D). The horizontal dashed line inside the box indicates the mean value, the horizontal solid line indicates the median, the upper border of the box indicates the upper quartile, the lower border of the box indicates the lower quartile, the upper edge of the box indicates the maximum value, and the lower edge of the box indicates the minimum value. NRS2002: Nutrition Risk Screening; PG-SGA SF: Patient-Generated Subjective Global Assessment-Short Form.


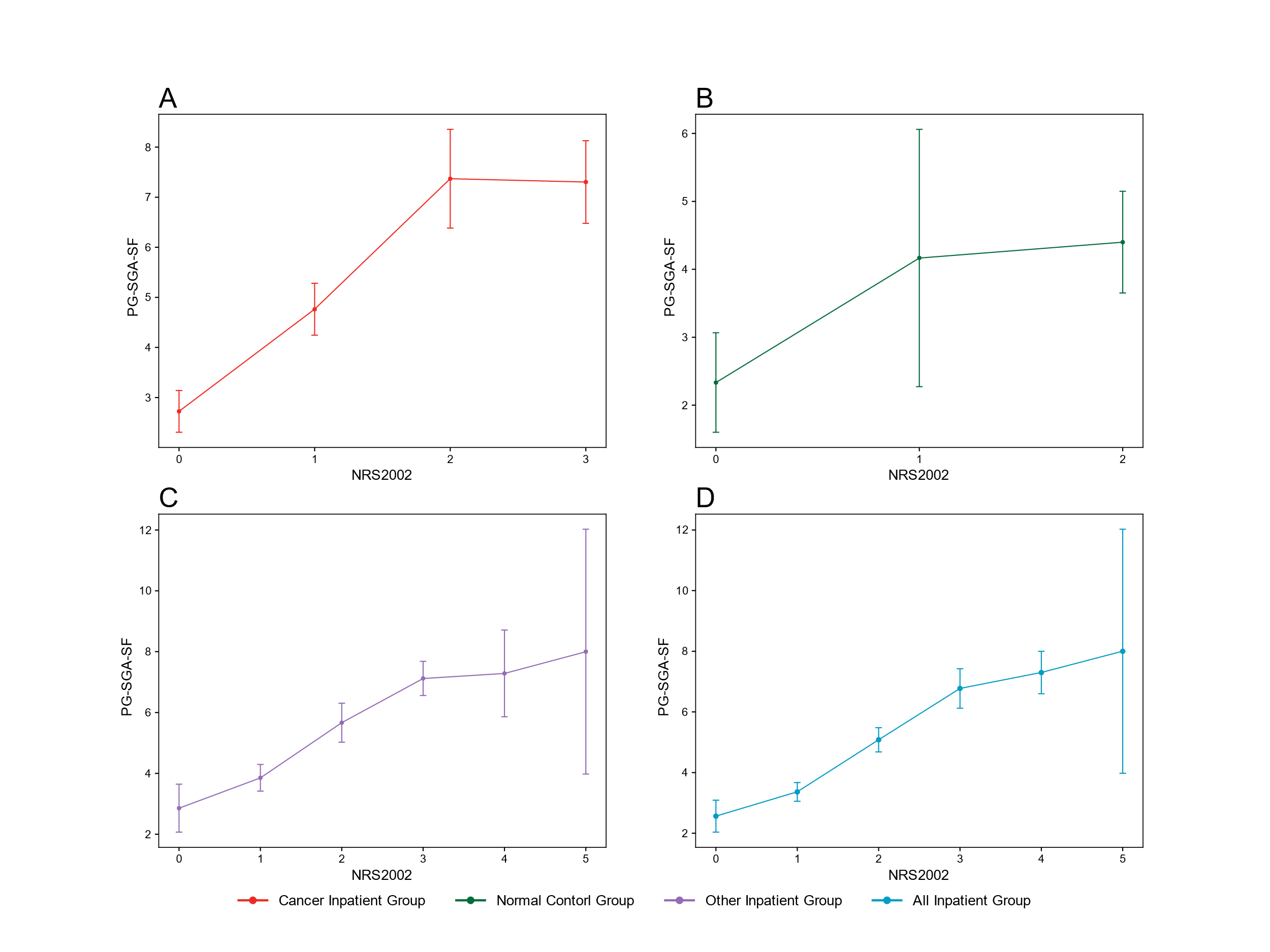


**Figure S2: Confidence error plot of NRS2002 and PG-SGA SF data for inpatient groups**

Error plot obtained from NRS2002 and PG-SGA SF for the four inclusion groups of Cancer Inpatient Group(A), Normal Control Group(B), Other Inpatient Group(C), All Inpatient Group(D). The dot in the figure is the mean of PG-SGA SF corresponding to NRS2002, and the distance from the upper and lower boundary of the error bar to the dot is the corresponding 95%. NRS2002: Nutrition Risk Screening; PG-SGA SF: Patient-Generated Subjective Global Assessment-Short Form; CI: confidence interval.


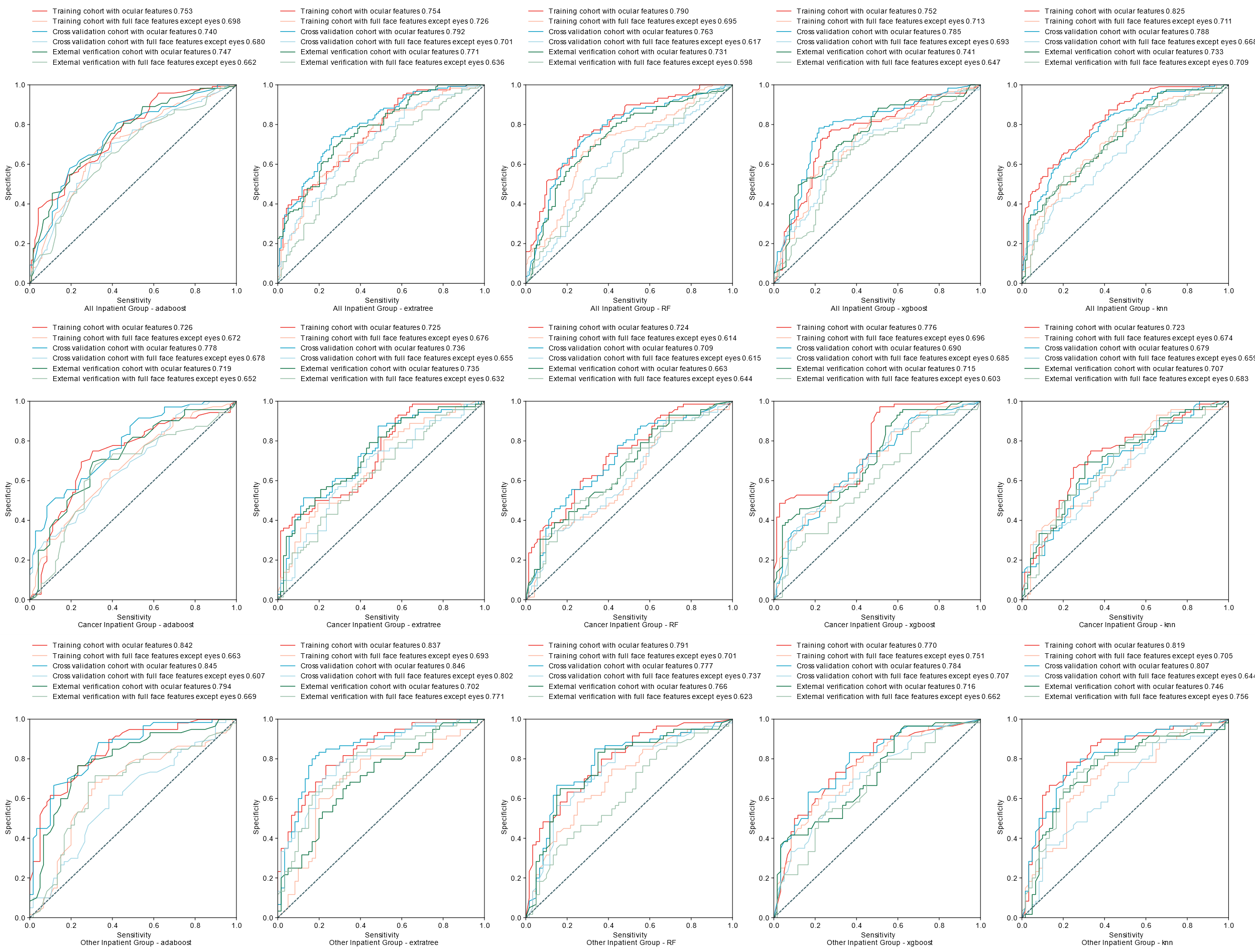


**Figure S3: AUC of different classification models to identify the nutritional status with different inpatient groups**

Five classifiers, including AdaBoost, Extratree, RF, XGBoost, and k-NN, are used in Cancer Inpatient Group, Other Inpatient Group and All Inpatient Group to classify and recognize nutrition for Ocular features and Full face features except eyes. The ROC curve was used to compare the five parameters of Accuracy, Sensitivity, Specificity, PPV and NPV. Compare the AUC results of the training cohort, cross validation cohort and external validation cohort using different features under the same classifier through the ROC curve. AUC is micro-AUC. ROC: Receiver Operating Characteristic; RF: Random Forest; AUC: Area Under roc Curve.


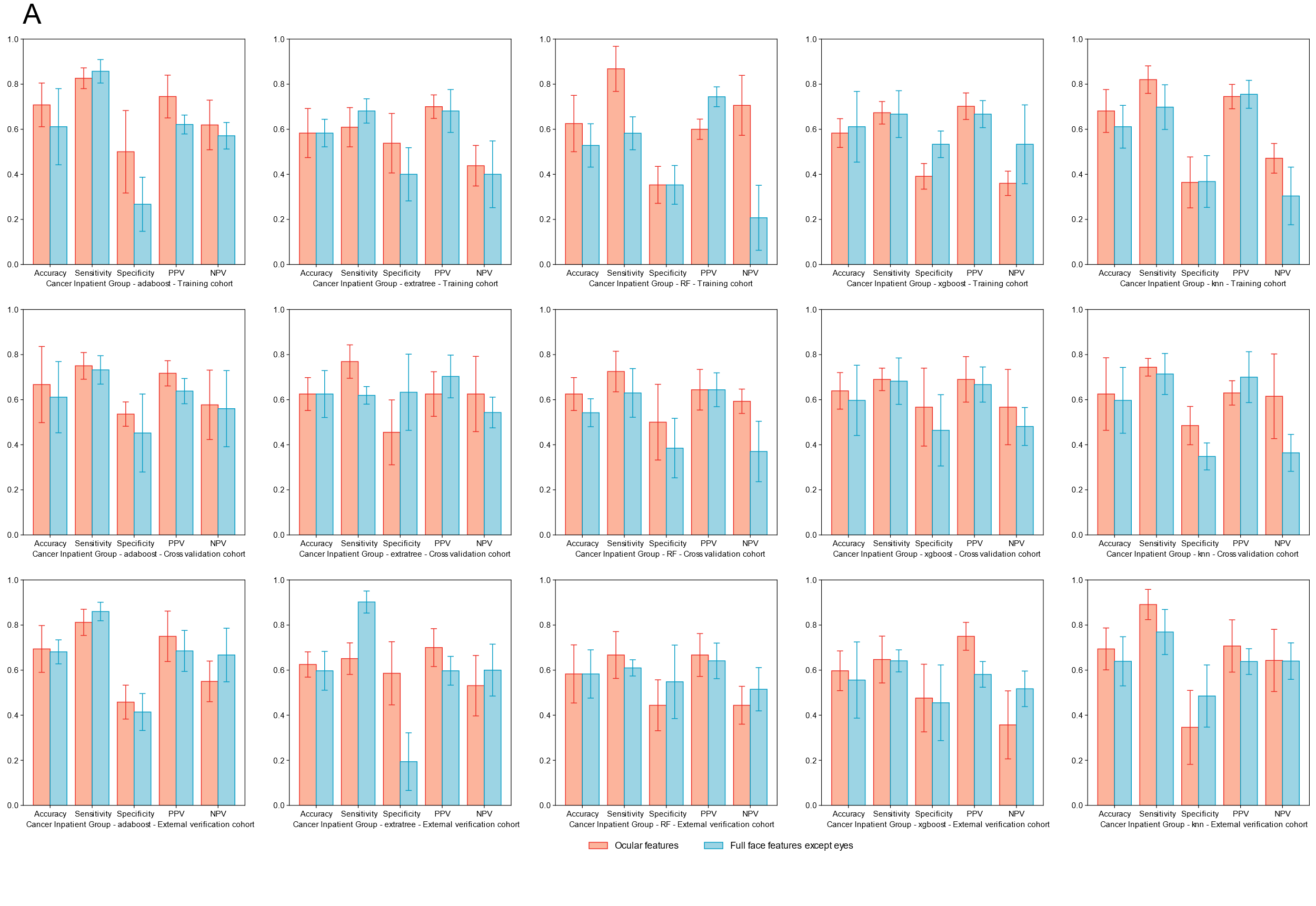


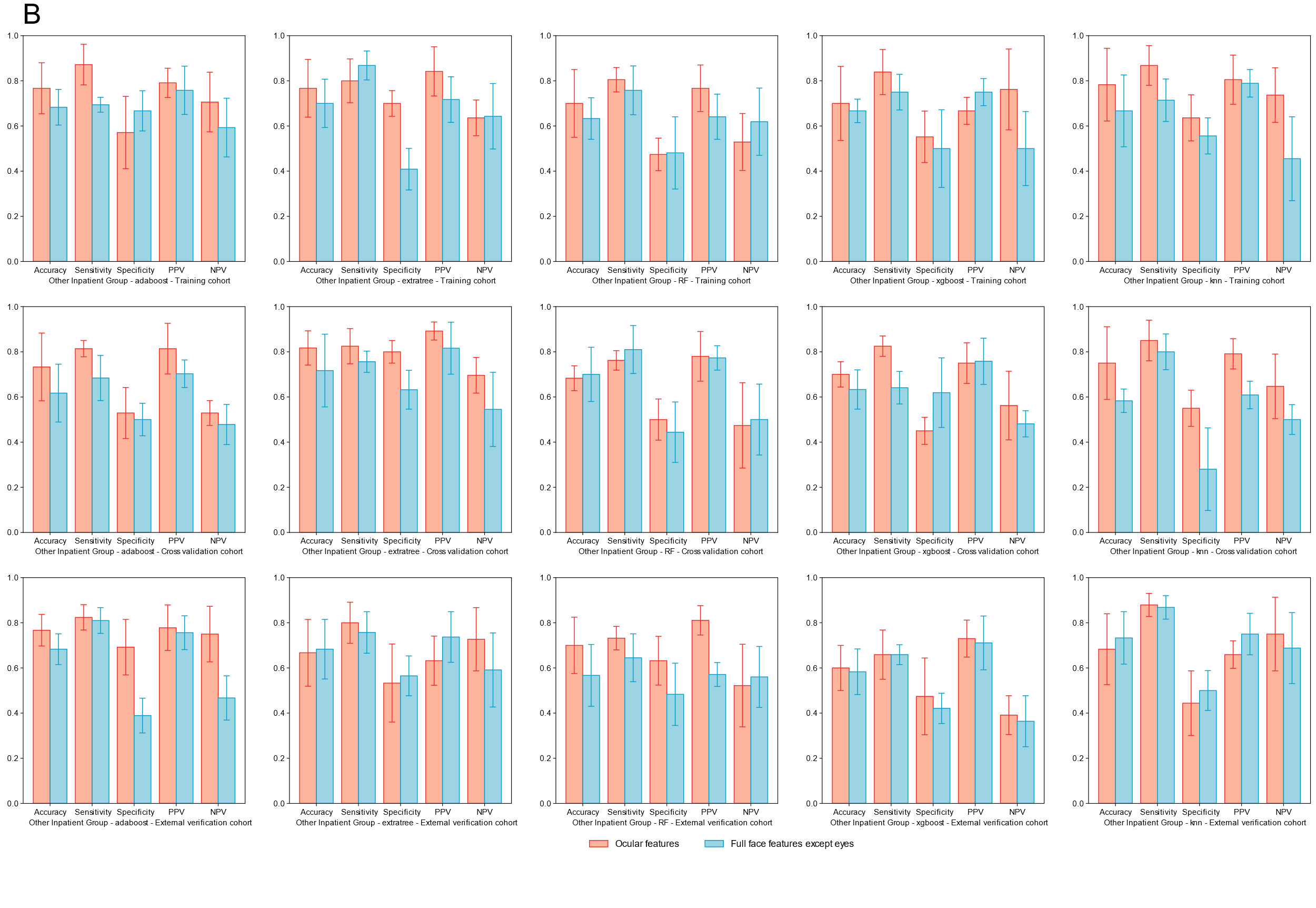


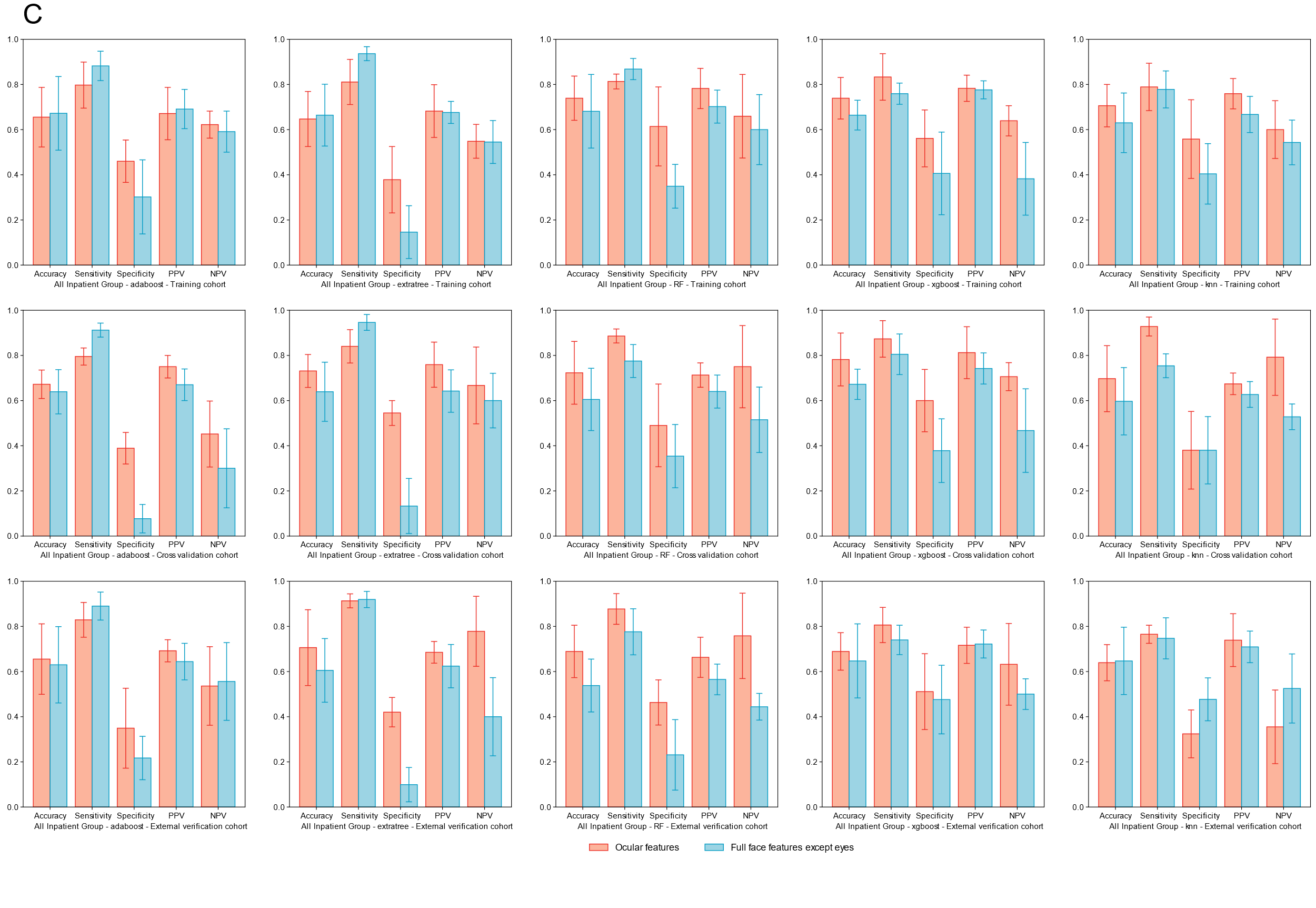


**Figure S4:** **Performance of different classification models to identify the nutritional status with different inpatient groups**

Five classifiers, including AdaBoost, Extratree, RF, XGBoost, and k-NN, are used in Cancer Inpatient Group (A), Other Inpatient Group(B) and All Inpatient Group(C) to classify and recognize nutrition for Ocular features and Full face features except eyes. The bar chart was used to compare the five parameters of Accuracy, Sensitivity, Specificity, PPV and NPV. bar represents the average value, and the distance between the upper boundary and the lower boundary of the error bar and bar is the corresponding 95% CI. RF: Random Forest; PPV: Positive Predictive Value; NPV: Negative Predictive Value; CI: Confidence Interval.

**
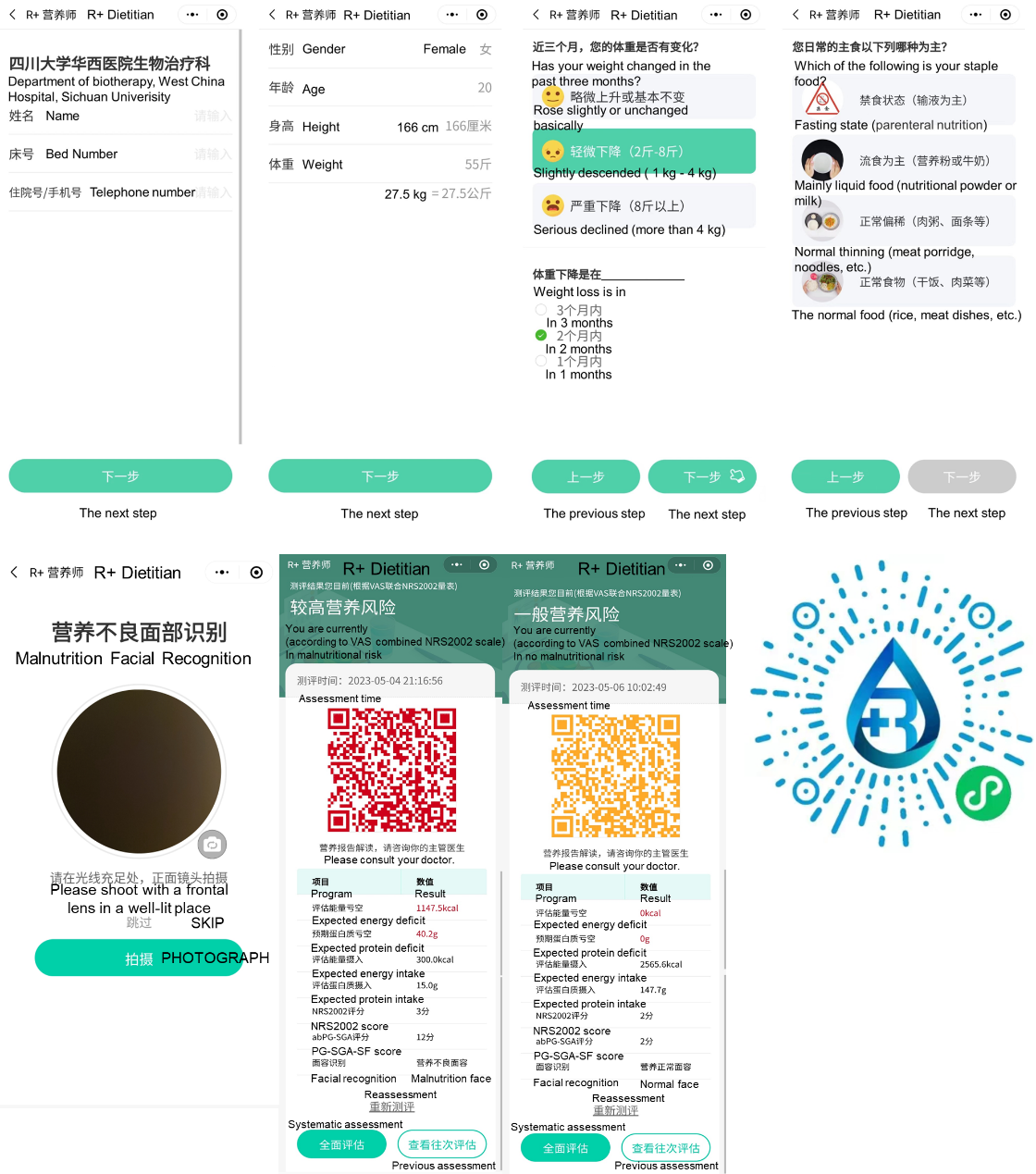
Figure S5: The procedure of the malnutrition screening system in mobile phone.** The device was developed based on various inpatients. Once collecting the basic clinical feature, malnutrition screening scale and facial photograph, the patients’ nutrition status will be shown on mobile phone or computer.


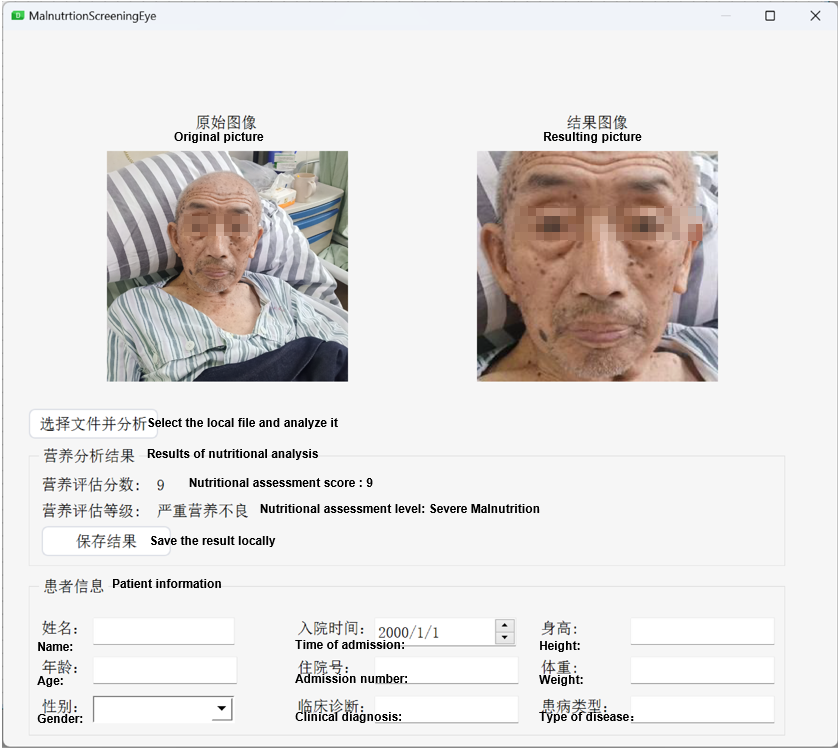


**Figure S6: The procedure of the point-of-care mobile solution for screening malnutrition in computer.** The device was developed based on various inpatients. Once collecting the basic clinical feature, malnutrition screening scale and facial photograph, the patients’ nutrition status will be shown on mobile phone or computer.
